# Harnessing the potential of human induced pluripotent stem cells, functional assays and machine learning for neurodevelopmental disorders

**DOI:** 10.3389/fnins.2024.1524577

**Published:** 2025-01-08

**Authors:** Ziqin Yang, Nicole A. Teaney, Elizabeth D. Buttermore, Mustafa Sahin, Wardiya Afshar-Saber

**Affiliations:** ^1^Rosamund Stone Zander Translational Neuroscience Center, Boston Children’s Hospital, Harvard Medical School, Boston, MA, United States; ^2^FM Kirby Neurobiology Center, Department of Neurology, Boston Children’s Hospital, Harvard Medical School, Boston, MA, United States; ^3^Human Neuron Core, Boston Children’s Hospital, Boston, MA, United States

**Keywords:** hiPSC, neurodevelopmental disorders, patch clamping, MEA, voltage imaging, calcium imaging, machine learning, translational research

## Abstract

Neurodevelopmental disorders (NDDs) affect 4.7% of the global population and are associated with delays in brain development and a spectrum of impairments that can lead to lifelong disability and even mortality. Identification of biomarkers for accurate diagnosis and medications for effective treatment are lacking, in part due to the historical use of preclinical model systems that do not translate well to the clinic for neurological disorders, such as rodents and heterologous cell lines. Human-induced pluripotent stem cells (hiPSCs) are a promising *in vitro* system for modeling NDDs, providing opportunities to understand mechanisms driving NDDs in human neurons. Functional assays, including patch clamping, multielectrode array, and imaging-based assays, are popular tools employed with hiPSC disease models for disease investigation. Recent progress in machine learning (ML) algorithms also presents unprecedented opportunities to advance the NDD research process. In this review, we compare two-dimensional and three-dimensional hiPSC formats for disease modeling, discuss the applications of functional assays, and offer insights on incorporating ML into hiPSC-based NDD research and drug screening.

## 1 Introduction

Neurodevelopmental disorders (NDDs) are a heterogeneous group of disorders that affect patients’ cognitive, communication, emotional, and motor development, with an onset at an early age (Mullin et al., 2013). NDD patients present with various symptoms, including language impairment, learning disabilities, seizures, and other neurological dysfunctions. Examples of the disorders include intellectual disability (ID), attention-deficit/hyperactivity disorder (ADHD), autism spectrum disorder (ASD), and epilepsy. Approximately 3% of young children worldwide have at least one NDD, and comorbidity (i.e., having more than one NDD) is common in these patients, which often results in missed diagnoses (Goldstein and Schwebach, 2004; Parenti et al., 2020; Francés et al., 2022; Bonti et al., 2024). While some symptoms associated with NDDs improve as the child gets older, or with early intervention, others persist into adolescence and adulthood, which leads to decreased independence, lowered occupational outcomes, and social disabilities (Hechtman et al., 2016; Halvorsen et al., 2019; Gidziela et al., 2023; Antolini and Colizzi, 2023). Progress to accelerate diagnosis of NDDs has been improved by advances in genetic testing, especially for severe and early-onset monogenic NDDs, but is still slowed by the lack of candidate biomarkers with high specificity and sensitivity to reliably detect the disorders before the onset of symptoms. Furthermore, therapeutic interventions are lacking to effectively treat NDDs across individuals, leading to mortality or lifetime disabilities in patients, high stress on the caregivers, and immense costs in healthcare and social welfare (Kularatna et al., 2022; Cortese et al., 2023). Since early diagnosis and intervention improve patient outcomes and alleviate the cost of following treatments, identifying and understanding the biological causes underlying the diseases are promising and important research directions.

Human induced pluripotent stem cells (hiPSCs) are a popular *in vitro* model for investigating disease mechanisms and testing therapeutic candidates (Anderson et al., 2021). hiPSCs are derived from patients and thus retain the human genetic backgrounds while being able to undergo various experimental manipulations. The pluripotency of the model allows researchers to differentiate hiPSCs into specific neuronal cell types (Takahashi et al., 2007; Zhang et al., 2013; Zhang et al., 2016; Yang et al., 2017; Ehrlich et al., 2017; Dolan et al., 2023) in 2-dimensional (2D) formats (Zhang et al., 2013; Qi et al., 2017) or into brain region-specific 3-dimensional (3D) organoids (Jo et al., 2016; Miura et al., 2020), which provides flexibility in experimental design depending on the research question. Functional assays (Belinsky et al., 2014) have been developed to characterize hiPSC models at various spatial and temporal resolutions. Additionally, the recent advancements in machine learning (ML) and its subset algorithms assist hiPSC disease models in further understanding NDDs (Trujillo et al., 2019). In this review, we compare various hiPSC model formats and discuss their advantages and limitations for investigating NDDs. We also provide systematic reviews on the phenotypic functional assays applied to hiPSC-derived cultures for several disorders and offer insight into their respective potential for therapeutic advancement. Finally, we will discuss the integration of ML into the hiPSC model to streamline the data analysis, mechanism investigation, and drug development process. To address the translational aspect of NDD research, the paper concludes with a discussion on the opportunities and challenges of hiPSC models with respect to bridging the gap between preclinical experiments and the clinic.

## 2 hiPSCs as an *in vitro* model for NDD disease study and drug screening

### 2.1 hiPSCs vs animal models

**FIGURE 1:**
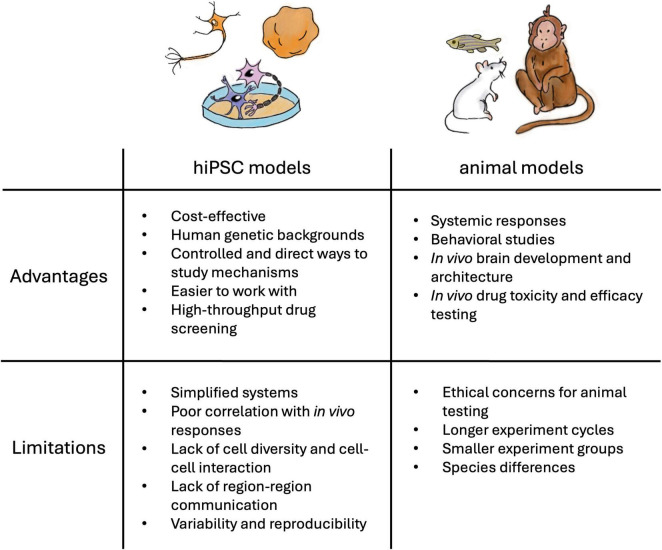
Advantages and limitations of hiPSC and animal models for NDD research and drug testing.

Animal models such as zebrafish, Drosophila, rodents, and non-human primates share highly similar genetic profiles with humans and have been used to model NDD phenotypes, including deficits in learning and memory, seizures, and hyperactivity (Sukoff Rizzo and Crawley, 2017; Damianidou et al., 2022). An advantage to using *in vivo* models is that they permit the investigation of connectivity between brain regions in the disease state. Furthermore, animal models offer the opportunity to study the complex interactions among different organ systems, physiological responses, and behavior (Figure 1). However, animals have different brain development milestones and do not exhibit the complex brain functions observed in humans (Gómez-Robles et al., 2024). Due to the lack of human-specific brain architecture and neuronal signaling mechanisms, studies have reported that the animal models fail to recapitulate the disease phenotypes or present minor symptoms (Goorden et al., 2007; Ehninger et al., 2008). For example, Kang et al. (2021) found that inhibition of the phosphoinositide 3-kinase pathway, rather than the metabotropic glutamate pathway identified in the fragile X syndrome (FXS) mouse model, rescued the neurodevelopmental defects in hiPSC-derived FXS forebrain organoids. Although animal models have largely contributed to our current knowledge, these models alone are insufficient to understand the underlying genetic and molecular mechanism of NDDs or to identify therapeutic targets for these disorders.

In contrast, hiPSCs present a promising opportunity to study NDDs *in vitro* in the context of human neurons (Figure 1). Derived from patient samples, hiPSC recapitulates the effects of genetic variations with the human genetic background. hiPSCs are reprogrammed back into an embryonic-like state and can be guided toward neural stem cells (NSC) and neural progenitor cells, which can give rise to central nervous system (CNS) neuronal subtypes to study brain development *in vitro* (Mariani et al., 2012). Gene editing technologies, such as CRISPR/Cas9, offer the possibility to generate corresponding isogenic controls (Hinz et al., 2019; Afshar-Saber et al., 2024a), reporters (Galiakberova et al., 2022), and other experimental manipulations (Puppo et al., 2021). However, issues related to variability and reproducibility have hindered preclinical findings from translating into drug discovery (Anderson et al., 2021). This represents a great challenge and needs to be continuously addressed. Toward this end, hiPSC maintenance and differentiation protocols, as well as recommendations for quality control procedures, have been established (Anderson et al., 2021; Ludwig et al., 2023; Sandoval et al., 2024), while commercialized cells and assay kits are available for trials and pilot experiments, making hiPSCs an advantageous model for conducting NDD research.

### 2.2 2D vs 3D hiPSC models

The 2D or monolayer hiPSC-derived culture is a well-established format for studying NDDs in specific cell types. hiPSCs can be differentiated into a reproducible and homogenous population of excitatory neurons (Zhang et al., 2013), inhibitory neurons (Yang et al., 2017), astrocytes (Zhang et al., 2016; Voulgaris et al., 2022), microglia (Abud et al., 2017; Dolan et al., 2023), and oligodendrocytes (Ehrlich et al., 2017; Martinez-Curiel et al., 2023) using small molecule combinations (Chambers et al., 2009; Qi et al., 2017; Cao et al., 2017) or transcription factors (Zhang et al., 2013; Ehrlich et al., 2017). The small molecule approach guides hiPSCs through *in vivo*-like neurogenesis, while the transcription factor approach bypasses the steps in between and produces target cell types rapidly. Additionally, 2D cell cultures allow easy access to measure the cellular and functional changes in the disease state. Marchetto et al. (2010) modeled Rett syndrome (RTT) using hiPSC-derived neurons and observed a decrease in synaptic formation, axon spine density, and neuronal activity of proband compared to the controls. Vijayalingam et al. (2020) examined the effects of CtBP1 mutation on neurodevelopmental delays through transcriptomic studies, consistent with the observed reduction in cell size and calcium activity in the patient hiPSC-derived neuronal cultures. In addition to disease studies, monolayer cultures can be the starting material for stem cell-based therapies (Damianidou et al., 2022). However, 2D cultures lack intercellular interactions with other types of cells and extracellular interactions with surrounding culture matrices. Studies also report shorter neurites (Chandrasekaran et al., 2017) and less mature networks in 2D systems compared to *in vivo* brains or cortical organoids (Woodruff et al., 2020).

Co-culture systems with two or more hiPSC-derived cell populations are one way to increase the heterogeneity of 2D models and to study intercellular interactions. Astrocytes regulate synaptic formation, modulate network activity, and provide metabolic support to neurons *in vivo* (Eroglu and Barres, 2010; Perea et al., 2014; Mederos et al., 2018). Plating hiPSC-derived astrocytes with excitatory neurons *in vitro* was reported to increase spontaneous activity in neurons and network synchronization (Tang et al., 2013; Ishii et al., 2017; Tukker et al., 2018; Hedegaard et al., 2020). Furthermore, co-cultures where specific genotypes and cell types are selected and plated together can be employed to interrogate the non-cell-autonomous impacts of disease-causing variation (Sun et al., 2023; Sharma et al., 2023; Parnell et al., 2023). For example, Supakul et al. (2024) observed tripartite synapse formation and an astrogliosis-like phenotype in neuron-astrocyte co-culture derived from a familial Alzheimer’s Disease patient, recapitulating the brain pathology that was not detected in the monoculture. In addition, co-cultures can be utilized to investigate the impact of alterations in cell population ratios that are observed in NDDs, including ASD (Lee et al., 2017; Culotta and Penzes, 2020; Vakilzadeh et al., 2024), schizophrenia (Gao and Penzes, 2015), Down syndrome (Zdaniuk et al., 2011) and ADHD (Bogdańska-Chomczyk et al., 2024). Importantly, the ratio of cell populations has been reported to affect the neuron development and activities *in vitro* even in control lines (Tukker et al., 2018; Ahtiainen et al., 2021; Parodi et al., 2023), but the consensus on plating density warrants further investigation. Therefore, while the co-culture system allows studying the interaction between cell populations of interest in a controlled laboratory setting, the plating ratio of different cell types should at least represent the *in vivo* state (e.g., a 3:1 ratio of excitatory-inhibitory neurons tested in (Parodi et al., 2023).

Additionally, 3D brain organoids are an alternative format to study NDDs. After being programmed into NSCs, the cells can self-aggregate into spheres and develop into cerebral organoids through “self-patterning” (Lancaster et al., 2013; Lancaster and Knoblich, 2014; Quadrato et al., 2017) or “directed patterning” in which a specific brain region is formed (Yoon et al., 2019; Whye et al., 2023). Single-cell RNA sequencing data of brain organoids generated using either approach reveals diverse cell types and populations similar to the corresponding regions *in vivo* (Velasco et al., 2019). In addition, the organoid model allows the study of early brain maturation (Gordon et al., 2021), as well as cell migration (Urresti et al., 2021) and network development and activity (Trujillo et al., 2019; Sharf et al., 2022). Several groups have utilized organoid models to study ASD (Urresti et al., 2021; Wang et al., 2022; Jourdon et al., 2023; Bury et al., 2024), FXS (Kang et al., 2021), Dravet syndrome (DS) (Yokoi et al., 2023), and Tuberous Sclerosis Complex (TSC) (Eichmüller et al., 2022), where the mixture of cell types present in the model allows for a better understanding of the developmental process and cell-cell interactions. A novel technique using organoids to uncover how developing neurons integrate into circuits of other brain regions is grafting, where mature cortical organoids are transplanted into animal models (e.g., rodent models) for studying neuronal development in an *in vivo* environment (Figure 1). Transplanted neurons have increased complexity in morphology and intrinsic firing properties compared to *in vitro* counterparts (Revah et al., 2022), but this approach can pose ethical considerations that need to be navigated (Hyun et al., 2022; Hoppe et al., 2023; Pas̨ca, 2024). Another popular approach is employing assembloids—where multiple spheroids are integrated—to study interregional cell migration, neural circuit formation, signal transduction, and cell-cell interaction *in vitro* (e.g., Andersen et al., 2020; Kim et al., 2024). For instance, Birey et al. (2017) observed the cell-autonomous defects in interneuron migration from the subpallial spheroids to cortical spheroids in the Timothy Syndrome assembloids compared to the controls, suggesting directions for future investigation and treatment target. Alternative methods to generate 3D hiPSC models include scaffold-based approaches using an extracellular matrix, bioreactors, or microfluidic chips (Huang et al., 2022). Nonetheless, the 3D model faces challenges such as the lack of nutrient delivery mechanisms to the core of mature organoids which lead to necrotic cores and lack of vascularization. These 3D models also suffer from limitations such as the inability to reconstitute complex brain architecture. Additional challenges related to scalability and reproducibility are greatly influenced, particularly when it comes to developing more intricate structures, as managing the growth and specialization of various cell types within multicellular or multi-tissue organoids is difficult (Urrestizala-Arenaza et al., 2024).

The choice to use either 2D or 3D cultures depends on the questions of interest, and studies have provided great details on the comparisons between the two formats (Liu et al., 2018; Damianidou et al., 2022). At the same time, groups have taken advantage of both systems to investigate the molecular and cellular mechanisms of NDDs. To study CDKL5 deficiency disorder (CDD), Negraes et al. (2021) cultured 2D hiPSC-derived cortical neurons to study the cellular morphology and synaptic formation of neurons while conducting electrophysiological recordings on the cortical organoids to investigate the network activity and synchronization. Brighi et al. (2021) measured the circuit function in FMRP-deficient hiPSC-derived cortical neurons using calcium imaging, and they examined cell diversity and development in 3D cortical organoids, demonstrating the role of FMRP in cell proliferation and network development. Therefore, each hiPSC model format provides opportunities to examine different properties and characteristics of NDDs *in vitro*.

### 2.3 hiPSCs as a high-throughput screening platform for drug discovery

Although the etiology of many NDDs has been linked to single gene variations, effective therapeutic targets to treat NDDs remains to be discovered and developed. Every year, pharmaceutical companies and research groups put tremendous effort into designing novel drugs based on the known pathological mechanism of the disorders. These candidates undergo multiple rounds of rigorous efficacy and safety tests before being approved for clinical trials, yet more than 90% of these attempts fail (Sun et al., 2022). CNS drugs in general have a higher failure rate than other systems, especially regarding clinical efficacy (Williams et al., 2022). Lack of efficacy in the clinic is due in part due to lack of target-relevant biomarkers in NDD patients, but also stems from lack of translation of targets found in preclinical studies. Therefore, to identify novel targets, filter out non-targeting compounds, develop drugs with minimal toxicity and side effects, and increase the possibilities of reaching clinics to help patients, a well-designed, optimized, and efficient preclinical model and drug screening platform are essential (Hughes et al., 2011). Important components of an ideal drug screening platform include: (1) a model with reproducible and representative disease phenotypes, (2) high-throughput assays for testing large volumes of drug candidates, (3) computational programs for analyzing the obtained data rapidly and accurately, and (4) translatable results to human clinical endpoints or biomarkers.

While *in vivo* models have been a popular platform for testing potential drug candidates for decades, the biological and genetic differences between humans and animals hinder translation to patients. There are also ethical concerns and restrictions on treating animals with chemicals (Kiani et al., 2022). In contrast, preclinical models based on hiPSCs emerge as a complementary and promising drug testing platform for NDD treatment. Besides their advantages for studying disease mechanisms, it is also easy to use the cell-based model to conduct long-term studies on drug effectiveness, dose concentrations, and cytotoxicity for early-stage drug discovery and validation, which would improve translational success. Examples of drug screening with 2D cultures include testing retigabine for ALS (Wainger et al., 2014) and antisense oligonucleotide (ASO) treatments for Angelman Syndrome (Dindot et al., 2023), which both moved to clinical trials. At the same time, several functional assays are well established for accurate and rapid readouts and analysis for drug screening on 2D cultures, which we will discuss later in this review.

Additionally, brain organoids have become an increasingly popular model for drug screening because the cell types and neuronal network in an organoid are more *in vivo*-like, and organoids can model the complex aspects of NDDs. For example, Chen et al. (2024) employed Timothy Syndrome cortical organoids, forebrain assembloids, and rat transplanted with proband organoids to test ASO treatments, which rescued the defects in calcium channel activation, interneuron migration, and morphology, showing the promising therapeutic strategy for the disorder. Additionally, there are commercial services for generating streamlined, reproducible organoids with low variability (e.g., StemoniX^®^ microBrain^®^ 3D Assay-Ready 384 Well Plates), which can be a viable alternative for conducting drug screening. Even though the technologies to rapidly characterize organoids are still in development, brain organoids will undoubtedly advance drug discovery for NDDs and complement *in vivo* testing (Giorgi et al., 2024).

## 3 Functional assays for hiPSC models

Studying cellular activity and neural network development is a critical component of NDD research to understand how the disorders affect neuron function. Investigating the effect of the disease genotype at the cellular and network level provides explanations for the clinical presentations of the disorders. For example, epilepsy, characterized by recurrent episodes of seizures (i.e., multiple hypersynchronous bursts from the neurons) (Stafstrom and Carmant, 2015), is a common neurological disorder and comorbidity with other NDDs (e.g., in 4–86% of ASD patients and 8–77% of ADHD patients) (Dunn et al., 2003; Keller et al., 2017). Imbalance between excitation and inhibition in cortical neurons–one of the possible neurological mechanisms of epilepsy–can be caused by ion channel dysfunction, ion homeostasis disruption, neurotransmitter dysregulation, or glial abnormalities (Bromfield et al., 2006; Shen et al., 2023). Modeling and monitoring the activity patterns *in vitro* allow researchers to investigate the cause of the disease phenotype and identify effective treatments. Therefore, it is critical to choose a functional assay that accurately measures the disease-relevant phenotypes when conducting NDD research.

Four established and widely applied methodologies for measuring neural activity are (1) patch clamping (Yajuan et al., 2012; Gao et al., 2021); (2) multielectrode array (MEA) (Obien et al., 2015; McCready et al., 2022); (3) voltage imaging (Knöpfel and Song, 2019), and (4) calcium imaging (de Melo Reis et al., 2020). These methods have been applied to hiPSC-derived neuronal cultures and contributed to the understanding of several NDDs, including FXS (Telias et al., 2015), TSC (Winden et al., 2019; Sundberg et al., 2021), DS (Doorn et al., 2023), Succinic Semialdehyde Dehydrogenase Deficiency disorder (SSADHD) (Afshar-Saber et al., 2024b), RTT (Dong et al., 2018), and more (Tables 1–4). Here, we focused on highlighting the respective advantages and new advancements in the context of their applications with hiPSC-derived cultures and potential as a high-throughput screening platform.

**TABLE 1:** How patch clamping was used to study NDD with hiPSC model.

NDD	Author	Model format	Configuration	Coupled with other functional assays?	Metrics used/reported	Key findings
Myoclonus dystonia (*SGCE* deficiency)	Sperandeo et al., 2023	2D excitatory cortical neurons (dual-SMAD inhibition)	Whole-cell	MEA and calcium imaging	Current clamp: RMP, Rin, Cm, τm, AP numbers, amplitude, half-width, rise time, fall time	*SGCE*-deficient neurons are intrinsically more excitable than the isogenic controls.
					Voltage-clamp: N/A	
Dup15q syndrome (*UBE3A* duplication)	Elamin et al., 2023	2D excitatory cortical neurons (dual-SMAD inhibition)	Whole-cell	Calcium imaging	Current clamp: Rin, AP amplitude, width, firing frequency, threshold, RMP, Cm	*UBE3A* duplication led to changes in intrinsic excitability and synaptic transmission of the hiPSC-derived neurons from patient samples, presenting as hyperexcitability. Early normalization of *UBE3A* expression at 6 weeks *in vitro* rescued the phenotype but was less effective at a later time point (16 weeks), suggesting that excess *UBE3A* is necessary but not sufficient to cause the phenotype.
					Voltage clamp: inward sodium current, outward potassium current, frequency and amplitude of sEPSC, sIPSC, mEPSC, and mIPSC, and interevent interval	
ASD (*NRXN1*α+/-)	Avazzadeh et al., 2021	2D excitatory cortical neurons (dual-SMAD inhibition)	Whole-cell	N/A	Current clamp: Rin, RMP, Cm, AP amplitude, rise time and slope, decay time and slope	*NRXN1*α+/- neurons showed altered sodium channel functions and hyperactivity compared to the control culture.
					Voltage clamp: ion channel currents	
FXS (*FMR1*-KO)	Susco et al., 2022	2D excitatory cortical neurons (NGN2 transcription factors + dualSMAD inhibition)	Whole-cell	N/A	Current clamp: RMP, Rin, τm, Cm, AP threshold, AP half-width, AP amplitude, AHP, f-I curves, max frequency	*FMR1*-KO neurons had premature and increased intrinsic membrane excitability and increased firing frequency compared to age-matched controls but showed no differences in synaptic transmission.
					Voltage clamp: sEPSCs amplitude, sEPSCs frequency, and percentage of cells with sEPSCs	
FXS (*FMR1*-KO)	Kang et al., 2021	Forebrain organoids (miniature bioreactor+dual-SMAD)	Whole-cell recording on organoid slices	N/A	Current clamp: RMP, Cm, Rin, AP firing frequency, first AP properties (amplitude, threshold, half-width, and rise time).	FMRP-deficient forebrain organoids showed higher firing frequency and larger potassium channel current, suggesting loss of FMRP could lead to an increase of potassium channels and hyperexcitability.
					Voltage clamp: ion channel currents	
FXS	Sharma et al., 2023	2D cortical neurons (from NPC generated with dual SMAD inhibition) and astrocytes (from APC using small-molecule approach)	Whole-cell	N/A	Current clamp: burst number, burst duration, firing pattern	Control neurons plated with FXS astrocytes or with FXS astrocyte-conditioned media displayed higher number of bursts but of shorter duration similar to the FXS neurons. On the other hand, FXS neurons co-cultured with control astrocytes or with control astrocyte-conditioned media showed similar activity as the control cells, suggesting the pivotal role of astrocytes in FXS phenotype. The neurons affected by FXS astrocytes also exhibited reduced persistent sodium current. Application of veratridine, a sodium channel opener, rescued the disease phenotype, while the potential astrocyte-derived candidate, S100ß, also showed the same effect.
					Voltage clamp: sodium current	
FXS	Zhang A. et al., 2022	2D GABAergic inhibitory neurons (small molecule)	Whole-cell	MEA	Current clamp: N/A	FXS and control GABAergic neurons showed similar miniature inhibitory postsynaptic currents, suggesting functional GABAergic synapses in both genotypes.
					Voltage clamp: miniature postsynaptic currents	
*STXBP1*-related disorder (*STXBP1*-RD)	van Berkel et al., 2023	2D excitatory cortical neurons (NGN2 transcription factors + dualSMAD inhibition)	Whole-cell (micro-islands)	Calcium imaging	Current clamp: N/A	No significant difference was observed between the healthy controls and *STXBP1*-RD neurons
					Voltage clamp: mEPSC amplitude and frequency, paired-pulse ratio, synaptic depression ratio,	
Angelman syndrome (*UBE3A* deletion)	Fink et al., 2017	2D cortical excitatory neurons (dual SMAD inhibition)	Whole-cell	Calcium imaging	Current clamp: RMP, AP maturation patterns (no AP, immature, single mature, and mature train), Rin, Cm, AP width, AP frequency, amplitude, FWHM	AS neurons exhibited depolarized resting membrane potential. AS and UBE3A-KO neurons displayed delayed firing maturation compared to isogenic controls. AS neurons also showed increased outward current throughout development but less than the isogenic controls, which could result in the immature firing activity. Both AS and UBE3A-KO neurons showed reduced synaptic activity and plasticity.
					Voltage clamp: inward sodium current, outward potassium current, sEPSC frequency and amplitude	
Angelman syndrome (*UBE3A* deletion)	Sun et al., 2019	2D excitatory neurons (transcription factor) 3D cortical spheroids (dual-SMAD + small molecules)	Whole-cell Whole-cell/whole mount	Calcium imaging	Current clamp: spike frequency, fAHP amplitude	Loss of *UBE3A* causes increased excitability with enhanced fAHP in 2D neurons and organoids compared to controls.
					Voltage clamp: big potassium channel current	
RTT (*MeCP2*)	Tang et al., 2016	2D astrocyte-assisted hiPSC/NPC derived-neurons	Perforated	N/A	Current clamp: GABA functional switch	*MeCP2*-mutated neurons showed an altered GABA functional switch, but IGF1, which treats glutamatergic deficits and increases KCC expression, can rescue the GABA functional switch.
					Voltage clamp: N/A	
RTT (*MeCP2*)	Pradeepan et al., 2024	2D cortical excitatory neurons (NGN2 transduction)	Whole-cell [data from Mok et al. (2022)]	MEA	Current clamp: RMP, firing rate, number of spikes	*MeCP2* null neurons displayed reduced rheobase compared to isogenic controls. *MeCP2*-deficient neurons also exhibited an increasing firing rate that peak at a low current and then declined with higher current input, while the isogenic controls had an increasing firing rate as the stimulus increased, suggesting a hyperexcitable disease phenotype.
					Voltage clamp: N/A	
RTT and CDD	Wu et al., 2022	excitatory neurons and glial cells from cortical organoid (patterning approach)	Whole-cell on cells migrating from the organoids	N/A	Current clamp: RMP, number of AP, AP amplitudes, half-width, threshold, depolarization, repolarization, rheobase	CDD organoids have increased intrinsic excitability, which could be caused by increased potassium and sodium current densities and faster sodium channel opening but not due to synaptic formation or altered glial activity. RTT organoids presented similar electrophysiological phenotypes as CDD organoids.
					Voltage clamp: Cm, Rin, voltage-dependent sodium and potassium channel current, current decay time, sEPSC	
EIEE13 (SCN8A-associated epilepsy)	Tidball et al., 2020	2D excitatory cortical neurons (dual-SMAD and Ngn1/NGN2 transcription factor)	Whole-cell	MEA	Current clamp: spontaneous action potentials, repolarization, AP amplitude, membrane potential after peak, RMP, Rin, Cm	EIEE13 neurons (dual-SMAD) showed variant-specific alteration in persistent and resurgent sodium channel currents compared to unrelated healthy controls, as well as early depolarizations and prolonged repolarizations. EEIE13 neurons differentiated using the transcription factor-based approach showed similar action potential shapes as those derived with the small-molecule-based method, with some differences in individual action potential metrics. Applying riluzole and phenytoin was shown to inhibit all spontaneous activity, which can be reversed through washout.
					Voltage clamp: sodium current, peak sodium current, persistent/peak sodium current, resurgent sodium current	
KCNQ2-associated epilepsy (R581Q variation)	Simkin et al., 2021	2D cortical excitatory neurons (NGN2 transduction + dual smad inhibition)	Whole-cell (automated patch clamp)	MEA	Current clamp: RMP, Rin, AP amplitude, half-width, fAHP, mAHP, sAHP, post-burst AHP	R581Q iPSC-derived neurons exhibit more depolarized RMP and higher Rin. However, the mutated neurons displayed slower AP repolarization at an early stage but faster AP half-width and enhanced fAHP over time compared to the control neurons. Administration of apamin, a SK channel antagonist, reversed the disease phenotype, and Chronic treatment of XE991, potassium blocker and M-current inhibitor, in control neurons replicated the observed phenotypes of R581Q neurons, suggesting an altered M-current and ion channel dysfunction.
					Voltage clamp: N/A	
Dravet syndrome (*SCN1A*-deficiency)	Van Hugte et al., 2023	2D cortical excitatory neurons (NGN2 transduction)	Whole-cell	MEA	Current clamp: RMP, number of AP, AP threshold, AP half-width, AP amplitude, AHP, firing rate, rise slope, rise time, max rise slope, decay slope, decay time, max decay slope, rheobase	Neurons with heterozygous loss of *SCNQ1A* showed altered intrinsic electrophysiological properties compared to controls, suggesting that the *SCNQ1A* deficiency affects excitatory neurons at single cell level.
					Voltage clamp: sodium current	

RMP, resting membrane potential; AP, action potential; Rin, membrane input resistance; AHP, after-hyperpolarization time; fAHP, fast AHP; sEPSC, spontaneous excitatory post-synaptic current; τm, membrane time constant; Cm, capacitance; FWHM, full width at half-maximum amplitude.

**TABLE 2:** How MEA was used to study NDD with hiPSC model.

NDD	Author	Model format	MEA manufacturer	Recording length	Media	Coupled with other functional assays?	Metrics used/reported	Key findings
FXS	Zhang A. et al., 2022	2D GABAergic inhibitory neurons (small molecule)	48-well CytoView LD-MEA (Axion Biosystem)	Every 5 days from day 47 to day 77	BrainPhys with supplements	Patch clamping	Mean firing frequency, max firing frequency, number of spikes per burst	FXS inhibitory neurons were more active compared to control neurons, and the GABAergic switch occurred later in the FXS culture.
*MEF2C* deficiency	Mohajeri et al., 2022	2d cortical excitatory neurons (NGN2 transduction)	48-well CytoView LD-MEA (Axion Biosystem)	Every 3 days from day 27 to day 60	BrainPhys	N/A	Weighted MFR, spike count, synchrony, oscillation	Neurons with loss of *MEF2C* displayed decreased network activity and synchrony, suggesting a disruption in the synapse formation.
TSC (*TSC2*-deficiency)	Winden et al., 2019	2D cortical excitatory neurons (NGN2 transduction)	48-well CytoView LD-MEA (Axion Biosystem)	Every other day for 25 days	Not reported	N/A	Weighted MFR, synchrony index	Neurons with loss of *TSC2* exhibited a hyperactive and hypersynchronized phenotype compared to isogenic controls. Chronic and early administration of Rapamycin, mTOR inhibitor, rescued the disease phenotype in TSC2 neurons, suggesting that mTORC1 hyperactivation might lead to epileptic activity.
RTT (*MeCP2* mutation)	Pradeepan et al., 2024	2D cortical excitatory neurons (NGN2 transduction)	12-well CytoView LD-MEA (Axion Biosystem)	Every week for 6 weeks	CM2 BrainPhys media	Patch clamping	Firing rate, mean burst firing rate, inter-burst-peak-intervals, network event duration, network event frequency, mini-burst frequency, % of bursting wells, mean number of bursts, reverberating super bursts	*MeCP2* null neurons exhibited a reverberating super burst (RSB) firing pattern and more active network activity compared to isogenic controls. EGTA-AM, a calcium ion chelator, decreased the RSB and the duration of the initiation network burst, suggesting that the altered firing pattern is calcium ion-dependent.
*CAPRIN1 haploinsufficiency*	Pavinato et al., 2023	2D cortical excitatory neurons (small molecule)	24-well LD-MEA (Multi Channel Systems)	Day 0 to 14 after neural differentiation	Not reported	Calcium imaging	Firing pattern, spike rate, spike count, burst count, burst duration	Neurons with heterozygous loss of *CAPRIN1* displayed decreased spontaneous firing rate and synchronization compared to controls.
KCNQ2-associated epilepsy (R581Q variation)	Simkin et al., 2021	2D cortical excitatory neurons (NGN2 transduction + dual SMAD inhibition)	12-well LD-MEA plate (Axion Biosystems)	Daily recording from days 15 to 31	Neurobasal media with supplement	Patch clamping	Number of bursts, number of spikes per burst, % of spikes that occur within a burst, MFR, ISI, burst frequency, burst duration, IBI	KCNQ2-mutated neurons started exhibiting spontaneous activity earlier than the isogenic control cells, while showing an increasingly phasic bursting pattern with more and higher percentage of spikes per burst and shorter interval between spikes. Applying apamin (a SK channel antagonist) and paxilline (a BK antagonist) to KCNQ2-mutated neurons rescued the network activity to the same level as the control neurons, while the control neurons with chronic XE-991 treatment, M-current inhibitor, suggesting the role of reduced M-current and ion channel dysfunction in altered network behavior in KCNQ2-mutated neurons.
Dravet syndrome(SCN1A-deficiency)	Van Hugte et al., 2023	2D cortical excitatory neurons (NGN2 transduction)	24-well LD-MEA (Multichannel systems, MCS)	DIV49	Not reported	Patch clamping	Normalized MFR, PRS, MBR, burst duration, network burst rate, network burst duration, number of high frequency bursts, bust spike rate	Neurons with heterozygous loss of *SCNQ1A* exhibited hyperactive, asynchronous network activity and mutation-specific firing pattern. Changing recording temperature to mimic the febrile seizure conditions led to altered neuronal network organization in GEFS+ neurons. In addition, anti-seizure medication rescued the disease phenotype in GEFS+ neurons but not in DS patients, showing a mutation-specific and clinically relevant phenotype *in vitro*.
EIEE13 (SCN8A-associated epilepsy)	Tidball et al., 2020	2D cortical excitatory neurons (NGN2 transduction)	96-well LD-MEA plate (Axion Biosystems)	From days 15 to 33 after doxycycline induction	BrainPhys with supplements	Patch clamping	Weight MFR, ISI, burst duration, % of spikes in network	EIEE13 neurons showed an increased burst activity and epileptiform-like firing pattern. Riluzole and phenytoin, drugs to inhibit persistent and resurgent sodium channel current, were shown to reduce the bursting phenotype.
N-acetyl neuraminic acid synthase (NANS) mutate	Bu et al., 2023	2D excitatory neurons (dual-SMAD inhibition) and organoid (self-patterning) slices	24-well LD-MEA Cytoview plate (Axion Biosystems)	DIV91	BrainPhys	N/A	Number of spikes, number of bursts, number of network bursts, synchrony index	Loss of NANS disrupted synapse formation and network activity in NANS-KO cortical neurons and cerebral organoids.
Myoclonus dystonia (SGCE mutation)	Sperandeo et al., 2023	2D excitatory glutamatergic cortical neurons (dual-SMAD inhibition protocol)	24-well Cytoview LD-MEA plates (Axion Biosystems)	Every 3 days from days 35 to 63 (cells plated at Day 30)	N2B27 (with Vitamin A)	Patch clamping and calcium imaging	Number of spikes, number of bursts, network busts, and synchrony index	SGCE-deficient neurons exhibit hyperexcitable network activity, compared to isogenic controls
16p11.2dup	Parnell et al., 2023	2D excitatory neurons (NGN2 transduction) and GABAergic neurons (Ascl1/Dlx2 transduction) co-culture	48-well CytoView LD-MEA (Axion Biosystem)	Every week from week 4 to week 7	Neurobasal media	Calcium imaging	MFR, synchrony index, network burst frequency	Co-culture of 16p11.2 dup (DUP) excitatory and inhibitory neurons and DUP excitatory neurons alone exhibited dysregulated and reduced network activity.
SSADHD (ALDH5A1-deficiency)	Afshar-Saber et al., 2024b	2D excitatory neurons (NGN2 transduction)	48-well CytoView LD-MEA (Axion Biosystem)	Every 2 days from DIV10 to DIV50	Not reported	Calcium imaging	synchrony index, MFR, average burst frequency, average burst duration, average number of spikes per burst, mean ISI within burst	Glutamatergic neurons with homozygous loss of *ALDH5A1* exhibited increased firing activity, reduced bursting frequency but more spikes per burst and longer burst duration compared to the neurons with heterozygous loss and isogenic controls, suggesting a hypersynchronous network development in the ALDH5A1-deficient neurons.
Kleefstra syndrome	Frega et al., 2019	2D excitatory neurons (NGN2 transduction)	24-well LD-MEA (Multichannel Systems)	Every 4 days from DIV7 to DIV 40	Neurobasal media with supplement	Patch clamping	MFR, MBR, burst duration, IBI, % spike out of burst,	KS neurons exhibited less frequency network bursts, longer burst duration, and an irregular firing pattern compared to the control neurons. NMDAR inhibition drove KS network activity toward the control.

MFR, mean firing rate; PRS, percentage of random spikes; MBR, mean burst rate; IBI, inter-burst interval; ISI, inter-spike interval; FXS, Fragile X Syndrome; TSC, Tuberous Sclerosis Complex; GEFS+, generalized epilepsy with febrile seizures plus.

**TABLE 3:** How voltage imaging was used to study neurological with hiPSC model.

Neurological disorders	Author	Model format	VI	Coupled with other functional assays?	Metrics used/reported	Key findings
EM	Alich et al., 2023	hiPSC-derived sensory neurons	Dark quencher GEVI (dqGEVI)	Patch clamping	Number of firing cells, ΔF/F traces, inter-event interval, inter-burst intervals, burstiness	EM sensory neurons are more active and more sensitive to increase in temperature compared to the controls.
ALS	Kiskinis et al., 2018	hiPSC-derived motor neurons	CheRiff and QuasAr2-mOrange2 (GEVI)	Patch clamping	Number of AP, spontaneous firing rate, ΔF/F traces, percentage of cells in depolarization block, AP waveform	The authors didn’t observe significant difference in neuronal activity between ALS and control motor neurons, which they attribute to the immaturity of the hiPSC-derived cell culture.
TSC	Williams et al., 2024	hiPSC-derived excitatory neurons (NGN2 overexpression)	CheRiff and QuasAr (GEVI)	N/A	Evoked activity: firing rate, first spike rate, last spike rate, AP waveform, AHP, AHP time, min. derivative, max. derivative, max. spike rate, frequency (high stimulation)	TSC-KO neurons showed hypoactivity under low stimulation but hyperexcitability under high-intensity stimulation, with narrower and deeper afterhyperpolarizations and steeper rise and decay time, compared to the isogenic controls

EM, erythromelalgia; ALS, amyotrophic lateral sclerosis; TSC, tuberous sclerosis complex; AP, action potential; AHP, afterhyperpolarization.

**TABLE 4:** How calcium imaging was used to study NDD with hiPSC model.

NDD	Author	Model format	GI	Coupled with other functional assays?	Metrics used/reported	Key findings
FXS	Brighi et al., 2021	2D cortical neurons (dual SMAD inhibition)	Fluo4-AM	N/A	Rise time, amplitude, event frequency, network synchrony, number of active cells, fluorescence intensity,	FMRP-KO neurons showed hyperexcitability, overactive network, and potential E/I imbalance compared to FMRP-WT neurons
SSADHD (ALDH5A1-deficiency)	Afshar-Saber et al., 2024b	2D cortical excitatory neurons (NGN2 transduction)	pLV- hSyn-jRCaMP1b (GECI)	MEA	Co-active neurons, event frequency, event amplitude	Glutamatergic neurons with homozygous loss of *ALDH5A1* displayed reduced event frequency, increased amplitude, and more co-active cells compared to isogenic controls and heterozygous loss of the gene, consistent with the MEA data, suggesting an enhanced network formation in the diseased culture.
RTT (R294X mutation)	Dong et al., 2018	2D astrocyte media-assisted astrocytes differentiated from neural progenitors	Fluo-4 (CSD)	Patch clamping on mouse astrocytes and brain slices	Frequency and amplitude of spontaneous, as well as ATP-, thapsigargin-evoked activity,	RTT astrocytes showed elevated frequency and amplitude in spontaneous and ATP-evoked activity. Using thapsigargin, a chemical inducing the release of Calcium ions from endoplasmic reticulum (ER), RTT astrocytes showed a higher storage of Calcium ions and faster leakage compared to the wildtype astrocytes.
RTT (*MeCP2* mutation)	Samarasinghe et al., 2021	Cortico-subpallial assembloid	AAV1 Syn:GCaMP6f virus	N/A	Amplitude, synchronization, number of microcircuit, number of neurons in each microcircuit	Cx+GE assembloids exhibited synchronized activity after BMI treatment but not in Cx+Cx assembloids, so Cx+GE fusion model were employed in RTT study. MeCP2 Cx+GE assembloids showed hypersynchrony and hyperactivity compared to control organoids. In addition, the assembloids with control Cx and MeCP2 GE showed the same hypersynchronous activity, while mutant Cx with control GE assembloids displayed similar activity as the unmixed control Cx+GE assembloids, suggesting the role of interneurons in the network dysfunction.
*STXBP1*-related disorder (*STXBP1*-RD)	van Berkel et al., 2023	2D excitatory cortical neurons (NGN2 transcription factors + dualSMAD inhibition)	Fluo-4AM (CSD)	Patch clamping	Burst events, burst frequency, average interburst interval, event area, event amplitude, fraction of participation in a burst event, network synchronicity, event duration, rise time, decay time	*STXBP1*-RD neurons have altered event frequency, burst characteristics, and network activity compared to the control cells with patient-specific differences.
TSC	Hisatsune et al., 2021	2D excitatory cortical neurons (dual SMAD inhibition)	Fluo-8AM (CSD) and fura-2 AM (CSD)	N/A	Number of active cells, event frequency, percentage of synchronous events, frequency of non-synchronous vs synchronous events, resting calcium level, KCl response	TSC2-deficient neurons showed highly synchronous activity compared to neurons with heterozygous loss of TSC2 and isogenic controls. Inhibiting spontaneous activity and stimulate activities using KCl showed that TSC2-deficient neurons had enhanced calcium ion influx upon membrane depolarization. Chronic treatment of rapamycin rescued the hyperactivity and decreased the expression of CACNA1D, a gene for calcium channel CaV1.3 subunit, in the TSC2-null neurons to the same level as the isogenic controls and the heterozygous loss, suggesting the role of calcium pump activity in the disease phenotype.
16p11.2dup	Parnell et al., 2023	2D excitatory neurons (NGN2 transduction) and GABAergic neurons (Ascl1/Dlx2 transduction) co-culture	Cal520-AM (CSD)	MEA	Normalized amplitude, normalized frequency, normalized number of synchronous events, normalized pairwise correction coefficient, average peak duration	16p11.2 duplication (DUP) excitatory neurons displayed reduced calcium event duration and increased calcium recovery time at single-cell level, while exhibiting decreased spontaneous activity frequency and network events compared to controls, suggesting a disruption in the synaptic formation. SCZ neurons also showed dysregulated calcium activity and reduced event frequency similar to DUP excitatory neurons, suggesting the duplication of 16p11.2 may contribute to the SCZ pathophysiology
Angelman syndrome (*UBE3A* deletion)	Fink et al., 2017	2D cortical excitatory neurons (dual SMAD inhibition)	Fluo-4AM (CSD)	Patch clamping	Number of calcium events, example calcium transients	AS and *UBE3A*-KO neurons showed decreased calcium events and synaptic plasticity compared to isogenic controls.
Angelman syndrome (*UBE3A* deletion)	Sun et al., 2019	3D cortical spheroids (dual-SMAD + small molecules)	Fluo-4AM (CSD)	Patch clamping	Interevent interval, calcium amplitude, event frequency, synchronization index	*UBE3A*-KO organoids showed early synchronized activity with increased frequency compared to control organoids
Myoclonus dystonia (SGCE mutation)	Sperandeo et al., 2023	2D excitatory glutamatergic cortical neurons (dual-SMAD inhibition protocol)	Fluo-4AM (CSD)	Patch clamping and MEA	Percentage activity, number of calcium transients, rise time, fall time, amplitude, and the interspike interval at three time points	SGCE-mutated cells are more active than isogenic controls, but have fewer calcium events and longer interevent intervals, suggesting a disruption in calcium-dependent activity and network functions
Dup15q syndrome (*UBE3A* duplication)	Elamin et al., 2023	2D neurons (dual-SMAD inhibition protocol)	X-Rhod-1 dye (CSD)	Patch clamping	Number of calcium transients	*UBE3A* duplicated cells showed increased spontaneous activity, compared to isogenic controls. ASO treatment that normalize the *UBE3A* expression reduced the event frequency, suggesting that *UBE3A* overexpression is necessary for the disease phenotype
*CAPRIN1* haplodeficiency	Pavinato et al., 2023	2D cortical excitatory neurons (small molecule)	Fluo-4 (CSD)	MEA	Fluorescence intensity	Neurons deficient of *CAPRIN1* displayed an increased calcium signal intensity, suggesting calcium ion overload in the cells.

### 3.1 Patch clamping

Patch clamping is a gold-standard, direct electrophysiological measurement of ion channel functions, which are proteins that regulate ion currents across the cell membrane. The technique was first invented in the 1970s as the loosely suctioned cell-attached mode (Neher and Sakmann, 1976), followed by whole-cell patch-clamp, with reduced background noise and increased temporal and spatial resolution to assess synaptic excitability in voltage mode or intrinsic excitability in current mode (Segev et al., 2016). Then, inside-out and outside-out configurations also became popular. The former permits studying the intracellular environment of the ion channel, and the latter focuses on investigating the properties of the ion channel isolated from the cell (Yajuan et al., 2012). Patch clamping is an excellent tool for studying action potential waveforms, ion channel current, and subthreshold membrane potential changes in neuronal cultures. While the traditional manual patch-clamp (MPC) technique is information-dense, it is time-consuming, labor-intensive, low-throughput, and requires extensive practice to obtain high-quality data (Yajuan et al., 2012). Consequently, the demand for overcoming these challenges prompts the development of automated patch clamping (APC) systems with higher throughput and lower skill requirements (Obergrussberger et al., 2016).

#### 3.1.1 Patch clamping data acquisition and analysis

**FIGURE 2:**
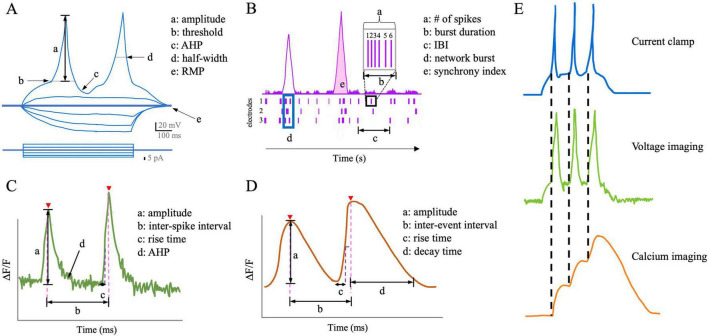
Schematic comparisons of the readouts of neuronal activity from each functional assay. **(A)** Schematic of action potential properties in current-clamp mode. AHP, afterhyperpolarization; RMP, resting membrane potential. **(B)** Schematic of common LD-MEA readouts about populational neuronal activity. IBI, inter-burst interval. **(C)** Schematic of voltage imaging readouts. AHP, afterhyperpolarization. **(D)** Schematic of calcium imaging readouts. **(E)** Compared to calcium imaging, voltage imaging better represents each action potential, despite slight delays in the signal decay.

Traditional MPCs have laborious setups before data acquisition (Leyrer-Jackson et al., 2019). Once set up, a differential interference contrast (DIC) microscope is essential to locate and identify the healthy cells for recording. Occasionally, fluorescent markers are used to identify specific cell types from a mixed population. Successful cell hunting takes time and expertise from human researchers using MPC, but also introduces bias from the investigator, while in APC, a robotic system executes most of the process. There are commercially available instruments for MPC (e.g., AxonMultiClamp from Molecular Devices, LLC) and APC (e.g., Syncropatch 768 PE from Nanion Technologies) that come with data analysis programs. Furthermore, interpreting patch clamping data depends on sample types (i.e., tissue slices or cultured cells), acquisition mode (i.e., voltage or current), and the configuration of patch clamping (i.e., cell-attached, whole-cell, inside-out, etc.). Comparative studies also showed differences in the data collected using MPC and APC (Franz et al., 2017). Nonetheless, conventional readouts of patch clamping include resting membrane potential, AP waveform, and after-hyperpolarization in the current clamp mode (Figure 2A).

#### 3.1.2 Application of patch clamping for studying NDDs using hiPSC models

Several genes associated with NDDs are linked to ion channel dysfunction (Lee et al., 2021), which is a common etiology of seizures (Lascano et al., 2016). Therefore, patch clamping is an ideal tool for studying NDDs linked to channelopathy and alteration in intrinsic activity. For example, Tang et al. (2016) applied patch clamping to a monolayer of hiPSC-derived neurons with *MeCP2*-mutation from RTT patients. They observed a disruption in the GABA functional switch in the disease culture, which was rescued by restoring K^+^-Cl^−^ cotransporter 2 through IGF-1 (i.e., insulin-like growth factor-1). Tidball et al. (2020) used hiPSC-derived neurons from patients with missense variations in *SCN8A* (i.e., a voltage-gated sodium channel gene linked to epilepsy). They observed persistent sodium channel current and altered AP waveforms in the patient line with whole-cell patch clamping. They then rescued the phenotypes in the disease line with riluzole (i.e., an FDA-approved persistent sodium current inhibitor), which is consistent with the decreased seizure frequency in patients after the drug administration. Combined with the advantages of the 2D hiPSC model, patch clamping examines the cellular mechanisms of NDDs and offers promising revenue for testing targeted therapeutic interventions.

Furthermore, patch clamping supports recording from co-culture systems because the researcher can selectively record from cells of interest. For example, to study the astrocyte-to-neuron communication in co-culture, Hedegaard et al. (2020) transduced hiPSC-derived astrocytes with Channelrhopsin-2, a light-gated ion channel commonly used in optogenetics, and hiPSC-derived neurons with mKate2, a fluorescence marker. Using whole-cell patch clamping, the authors observed that stimulation of astrocytes increased the spontaneous excitatory post-synaptic currents (sEPSCs) in neurons compared to the sEPSCs before stimulation, showing the effect of astrocytes on network activity. When examining the effect of astrocytes on neuronal excitability in FXS, Sharma et al. (2023) mixed hiPSC-derived healthy neurons, healthy astrocytes, FXS-neurons, and FXS-astrocytes in different cultures, and observed that FXS-astrocytes induced abnormal bursting phenotypes in control neurons, suggesting that astrocytes play a significant role in the disease mechanism. Pre-clinical studies of FXS have focused on neurons, with the role of glia remaining largely underexplored so in this study, the authors designed the previously described co-culture system to study the non-cell-autonomous effect in NDDs in combination with patch clamping thereby suggesting a framework for exploring new therapeutic strategies aimed at human neuron-glia interactions.

In addition, patch clamping has been applied to organoid models to investigate ion channel functions and electrophysiological properties as the neurons mature in an *in vivo*-like developmental sequence and environment. Wu et al. (2022) generated CDD and control cortical organoids. Using whole-cell recordings on cells migrating from the organoids, they detected an increase in intrinsic excitability and an alteration of voltage-gated ion channel functions in the CDD organoids. They also examined RTT organoids and observed the same firing patterns, suggesting a convergent mechanism between the two disorders. Sun et al. (2019) generated 2D and 3D hiPSC models of Angelman syndrome. Using whole-cell/whole-mount patch clamping, they found that dysfunction of Big Potassium channels likely caused the increase in AP firing frequency and the fast component of after-hyperpolarization, which results in the hyperactivity phenotypes in both model formats. Other studies also recorded from organoid slices (Pasca et al., 2015), dissociated cells (Gomes et al., 2020), and intact organoids (Landry et al., 2023). However, each recording method has drawbacks and fails to take full advantage of the 3D model. For example, dissociating organoids into 2D monolayer causes stress on the neurons and risks losing the network formed in the 3D culture while whole-mount recording only allows access to the exterior cells and cannot reach and investigate the interior of the organoid (Sandoval et al., 2024). The intact organoid recording protocol developed by Landry et al. (2023) claimed to record the electrophysiological and morphological features of cells from both the surface and sub-surface of the organoid with an additional step of clearing, which could be a viable option for investigating deeper layers of organoids.

### 3.2 Multielectrode array (MEA)

Multielectrode array (MEA), or microelectrode array, is an extracellular electrophysiological measurement of neuronal activity through direct contact between the cultured cells and the recording electrodes. It is a non-invasive technique with high temporal resolution that allows long-term investigation of cellular behaviors and network development, including firing patterns and synchronization. Low-density MEA (LD-MEA), such as Axion Biosystem Maestro Pro, has more wells on a plate (e.g., 6-, 12-, 24-, 48-, and 96-well) but fewer electrodes (e.g., 8–64 electrodes) in each well, so it accommodates more conditions and offers a bigger sample size at the cost of spatial resolution. On the other hand, high-density MEA (HD-MEA) has more electrodes covering each well, but fewer wells on one plate. For instance, MaxWell MaxOne contains up to 26,400 electrodes per well with at most 6 wells on a plate. Therefore, HD-MEA measures sub-cellular details (e.g., axon tracking), cellular activities (e.g., action potentials), and network connectivity (e.g., bursts and synchronization), but at a lower throughput to test fewer conditions.

#### 3.2.1 MEA data requisition and analysis

Commercially available 2D MEA plates with their compatible MEA machines and software for data acquisition and analysis (e.g., Axion Biosystems, MaxWell Biosystems) streamline the experimental setup. Nonetheless, several factors must be considered when designing and running experiments with MEA, including culturing conditions, experimental design, and data analysis, as reported by Mossink et al. (2021). Additionally, studies have reported using various culture media, including CM2 media (Mossink et al., 2021), BrainPhys (Quraishi et al., 2019; Graef et al., 2020), and DMEM/F12 with supplements (Nageshappa et al., 2016), but future studies should investigate the effect of media on functional activity, which would be applicable to all of the functional assays described in this review. In addition, temperature and CO2 concentration setting during the MEA recording change neuronal activity (Van Hugte et al., 2023). McCready et al. (2022) also provided a detailed overview and recommendations on the use of MEA, but future research is warranted to test how different culturing conditions affect the overall functional activity.

In terms of data analysis, a typical recording session generates various metrics to characterize the activities (e.g., bursting rate, bursting frequency, etc.) (Figure 2B), but it can be challenging for beginners to understand all the parameters in the output and for researchers to decide what metrics to report in a paper. There is also no consensus on the criteria to exclude inactive wells from the analysis, which could result in selection bias. The field will benefit from a standardized procedure of MEA.

#### 3.2.2 Applications of MEA for studying NDD disease mechanisms using hiPSC models

LD-MEA has been used extensively with 2D hiPSC cultures in long-term studies of network activity and synchronization in various NDDs. Winden et al. (2019) plated hiPSC-derived cortical neurons from TSC patients on 48-well MEA plates to study the neuronal activity over 25 days and observed an increase in the spontaneous activity and synchrony consistently in the *TSC2*-deficient line, which recapitulated the hyperactivity and hypersynchrony of seizures in patients. To study SSADHD, Afshar-Saber et al. (2024b) generated glutamatergic neurons with hiPSCs from patients with biallelic loss of *ALDH5A1*, sex-matched parental controls with monoallelic loss of *ALDH5A1*, and CRISPR-corrected control of the patient hiPSCs. Using MEA to monitor activities from day *in intro* 10 to 50, they found that the neurons with homozygous loss of *ALDH5A1* formed early synchronization, longer bursting, and faster spikes within each burst compared to the parental and the CRISPR-corrected controls, suggesting an altered firing behavior of glutamatergic neurons caused by the variant. Thus, longitudinal LD-MEA recordings provide useful insights into the development of network firing patterns and connectivity over time to elucidate the effects of disease on neural circuit behavior.

In addition, multiple NDDs are reported to exhibit deviated ratios of cell populations (Zdaniuk et al., 2011; Gao and Penzes, 2015; Lee et al., 2017; Culotta and Penzes, 2020; Vakilzadeh et al., 2024; Bogdańska-Chomczyk et al., 2024). For example, the imbalance between excitatory neurons and inhibitory neurons has largely been thought to play a role in the etiological mechanisms of ASD. Parodi et al. (2023) mixed hiPSC-derived glutamatergic and GABAergic neurons at different ratios with astrocytes on LD-MEA plates and monitored the activities for 98 days. Interestingly, they observed that an extreme imbalance of the excitatory/inhibitory (E/I) ratio (i.e., no GABAergic or no glutamatergic neurons) resulted in few, if any, bursting activity and synchronization, while the presence of inhibitory neurons at any ratio in the co-culture increased bursting activity and network connections, suggesting the role of inhibitory neurons in circuit function and development. However, while the MEA is a useful tool to measure total network activity, it fails to detect cell type-specific activity because the electrodes indiscriminately measure activities from all the cells in contact. There are commercial packages to physically separate cell populations in the same well (e.g., well divider insert by Axion Biosystems), but either the two populations are not fully connected, or the process of removing the divider for building a network can introduce complications or damage the cells. Future advancements in MEA technology are encouraged to detect the functional activity of distinct cell types in co-culture.

Furthermore, MEA can measure network oscillations in a mature brain organoid model to study *in vivo*-like brain development *in vitro*. Trujillo et al. (2019) carried out a comprehensive, long-term study of cortical organoids by growing them on 2D LD-MEA plates. They characterized the development of the network activity of the organoids, which highly correlated with the EEG data from preterm infants. However, due to the flat surface design, it is challenging to ensure that the organoids are contacting the recording electrodes. Additionally, the results only reflect the activity from the outer cells and fail to capture the complex network activity inside the organoids. Therefore, some groups plate organoid slices on MEA plates to study the interior network activity. For example, Bu et al. (2023) used slices of control and N-acetylneuraminic acid synthase (NANS) mutated cortical organoids on MEA plates and observed a decrease in network bursting and synchronization in the *NANS*-mutated organoids. On the other hand, comparative studies have shown that 2D HD-MEA provides a more accurate measurement of the interior cortical organoid activity than LD-MEA (Muzzi et al., 2023). Other groups also developed custom-designed MEA plates (e.g., 3D MEA or mesh MEA) to measure the interior activity of organoids, but these plates require more human labor and have lower throughput (Quadrato et al., 2017; Muzzi et al., 2023).

### 3.3 Imaging-based functional assays

Besides patch clamping and MEA, imaging-based methods, including voltage and calcium imaging, are prevalent options to measure neuronal activity and have been utilized for over 40 years (Cohen et al., 1974; Tsien, 1980). Both methods are non-invasive and utilize chemically synthesized or genetically encoded indicators which emit fluorescence signals in response to voltage changes or calcium influx during action potential events. Signals are recorded by a fluorescence microscope, which provides information on the activity of individual neurons as well as the network connectivity within the field of view. Depending on the indicators, a cell culture plate can be used for long-term studies over months. Detailed description and discussion of the methods are covered in other review papers (Braubach et al., 2015; Zlatic et al., 2021). We will briefly discuss the advantages and limitations of each method and focus on each has been applied to the study of NDDs using hiPSC models.

#### 3.3.1 Voltage imaging

Voltage imaging is the direct measurement of the changes in membrane potential through quantification of fluorescence intensity emitted from voltage indicators (VIs). This method has excellent spatial and temporal resolutions for visualizing electrical activities at the single-cell level from a population of cells that supports long-term investigation. VIs are able to detect sub-threshold activity of an action potential on a scale of milliseconds due to the sensitivity to changes in membrane potential.

##### 3.3.1.1 Voltage indicators (VIs)

Two main kinds of VIs are voltage-sensitive dyes (VSD) and genetically encoded voltage indicators (GEVIs). VSDs bind to the cell membrane indiscriminately, emit bright fluorescence signals in response to changes in membrane potential, and are often single use (i.e., need to be added before each recording session). Small molecule VSDs have great signal-to-noise ratio and excellent temporal resolution (Lippert et al., 2007). On the other hand, GEVIs are expressed by cells through transduction or transfection, which makes them more stable and durable, but consequently have dimmer fluorescence and lower signal-to-noise ratio than VSDs. However, GEVIs can target specific cell types through the design of promoters. The comparisons between these VIs and limitations are summarized in other review papers (Peterka et al., 2011; Kulkarni and Miller, 2017; Kannan et al., 2019; Aseyev et al., 2023), and several recent developments also aim to tackle the limitations of these VIs (Beck and Gong, 2019).

##### 3.3.1.2 Voltage imaging acquisition and analysis

Capturing the rapid changes in action potential requires a camera that can perform sub-millisecond recordings, which inevitably means a high exposure rate and risks of phototoxicity. One cannot instead lower the exposure rate, which makes the results less representative of the actual firing event. Another limitation of voltage imaging is cell segmentation when analyzing the recording. Whether chemically synthesized or genetically encoded, the VIs are located in the membrane, making it challenging to differentiate individual cells. Nonetheless, voltage imaging remains an excellent tool for optically profiling intrinsic neuronal behaviors (Hochbaum et al., 2014; Figure 2C).

##### 3.3.1.3 Applications of voltage imaging for studying NDD disease mechanism using hiPSC models

Voltage imaging does not yet have a wide application in NDD research. It has been used to measure the spontaneous activity of hiPSC-derived sensory and forebrain neurons from erythromelalgia (EM, i.e., a rare vascular pain disorder) patients (Alich et al., 2023). In the study, Alich and colleagues observed that EM sensory neurons from patient hiPSCs exhibited an increased bursting firing pattern compared to the sporadic firing pattern in the control cells. The EM sensory neurons also had a significant increase in firing rate in response to a mild rise in ambient temperature. The group also applied voltage imaging to the EM co-culture of glutamatergic and GABAergic neurons and detected a highly synchronized network connection. Other research groups would benefit from utilizing the VIs more often, particularly to quantify activity with high spatial and temporal resolutions.

In addition, voltage imaging was combined with optogenetics technique as the all-optical electrophysiology method, or “Optopatch”, to record both simultaneous and perturbed activity of cells, presenting a high-throughput method for studying ion channel functions (Hochbaum et al., 2014; Puppo et al., 2021). Kiskinis et al. (2018) applied this method to hiPSC-derived motor neurons of control and *SOD1*-mutation for amyotrophic lateral sclerosis (ALS). They found that the *SOD1*-variant motor neurons had higher spontaneous activity at no-to-low stimulation but lower activity when stimulated with high intensity, suggesting a disruption in neuronal firing functions. Groups have also used this approach on hiPSC-derived TSC-deficient neurons and observed increased repolarization in the waveform, spontaneous hypoactivity, and a hyperactive phenotype under high-intensity simulation (Williams et al., 2019; Williams et al., 2024). Multiple proof-of-concept studies have shown that multisite voltage imaging allows the study of signal propagations, AP travel speed, network connectivity, and voltage waveform (Jin et al., 2012; Gu et al., 2014; Milosevic et al., 2020; Walker et al., 2021).

One reason why the use of VIs is limited in research could be due to the restriction of the VIs to the membrane, which poses a technical challenge for recording. Additionally, the promotors of GEVIs often result in non-specific transfection and thus poor expression levels of GEVIs (Alich et al., 2023). Nonetheless, the assay can be a promising alternative to patch clamping with higher throughput and minimal invasiveness.

#### 3.3.2 Calcium imaging

Calcium imaging is a popular optical functional activity assay for *in vitro* studies, which is based on the principle that intracellular calcium ion level increases during a firing event. Similar to voltage imaging, this method also allows for long-term recordings at the single-cell level with minimal technical requirements.

##### 3.3.2.1 Calcium indicators (CIs)

Calcium indicators (CIs) detect the concentration of free calcium ions in the cytosol and emit fluorescence signals that can be measured by a microscope (Chen et al., 2013). Two main forms of calcium indicators are calcium-sensitive dye (CSD) and genetically encoded calcium indicator (GECI). CSDs are more efficient at detecting low-frequency, individual action potentials than GECIs (Tada et al., 2014), but all small molecule dyes share the same limitation in that they are not specific to one cell population and are not suitable for long-term experiments. Notably, GECIs have high signal-to-noise ratio and sustained expression in culture (Pologruto et al., 2004; Chen et al., 2013). GECIs can also be expressed in different intracellular compartments, such as the endoplasmic reticulum (Suzuki et al., 2014; Henderson et al., 2015) and mitochondria (Kanemaru et al., 2020), thus making them suitable for studying calcium flux in specific regions of the cell. Different families of GECI proteins are listed and discussed in other review papers (Whitaker, 2010; Suzuki et al., 2016).

##### 3.3.2.2 Calcium imaging data acquisition and analysis

A fluorescent microscope is required for acquiring calcium imaging data. Since calcium indicators have slower kinetics, the requirement on the camera and exposure time is lower than voltage imaging, which reduces the risk of phototoxicity. There are open-source and commercial calcium imaging acquisition and analysis platforms available for conducting studies using calcium imaging (e.g., NeuroPlex from RedShirtImaging, Mesmerize (Kolar et al., 2021), etc.).

However, due to the slow dynamics of calcium influx, fluorescence signals from calcium indicators have longer signal decay than electrophysiological measurements (Zhu et al., 2021; Figure 2D). The calcium transient peak is not linear (i.e., fluorescence from multiple electrical events can summate to one large peak if the electrical events occur in temporal proximity), making calcium imaging an ideal tool for investigating the pattern of neuronal activities (Figure 2D), but not the properties of individual action potentials during a firing burst (Pologruto et al., 2004; Whitaker, 2010; Ali and Kwan, 2020). Studies also report that certain calcium indicators could alter the morphology and physiological activity of culture (Gasterstädt et al., 2020), while other studies did not find such effects (Võfély et al., 2018), which should be considered when choosing the calcium indicator for the experiment.

##### 3.3.2.3 Applications of calcium imaging for studying NDD disease mechanisms using hiPSC models

Calcium imaging has provided useful information about network activity, synchrony, and development, as well as calcium dynamics of hiPSC-derived 2D models for NDD studies. Avazzadeh et al. (2019) found that hiPSC-derived 2D neuronal cultures with the *NRXN1α^+/–^* genotype (i.e., the most common rare genetic variation shared by multiple NDDs) exhibited increased calcium transients (i.e., amplitude, frequency, and duration), which was validated in another study that found impaired voltage-gated sodium, potassium, and calcium channel functions using patch clamping (Avazzadeh et al., 2021). When investigating TSC, Hisatsune et al. (2021) observed a hypersynchronous spontaneous activity in *TSC2*-null neurons compared to neurons with monoallelic loss of *TSC2* and isogenic controls. Using TTX to inhibit spontaneous activity and KCl to depolarize neurons, the authors found that the TSC2-deficient neurons displayed the highest increase in calcium ion influx. In addition, chronic treatment of rapamycin, an mTOR inhibitor, rescued the hyperactive phenotype and decreased the *CACNA1D* expression in the *TSC2^–/–^* neurons, suggesting an interaction between mTOR and calcium signaling in TSC pathology. Therefore, calcium imaging is a critical tool in studying disease mechanisms that allows researchers to visualize functional and calcium-related activities.

In addition, calcium imaging is advantageous for measuring the activity of co-culture and organoid systems of NDDs, thanks to the variety of fluorescence markers available for labeling multiple cell types or cell compartments. The technique has been applied to neuron-astrocyte (Kuijlaars et al., 2016) and microglia-motor neuron (Vahsen et al., 2022) co-culture systems and has the potential to benefit other combinations, such as excitatory-inhibitory neuron co-culture. In terms of the application to 3D brain organoids, a common way for recording is to use organoid slices. Rylaarsdam et al. (2024) performed calcium imaging on organoid slices of PACS2 syndrome at days 40 and 80 and observed the maturation of the network over time but no significant difference between the control and proband activities, which they attributed to the lack of GABAergic interneurons in the organoids. Notably, slicing could interrupt the neurons and neurites in the organoid, which could confound the recording results. On the other hand, Samarasinghe et al. (2021) generated cortical organoids with excitatory neurons and ganglionic organoids with inhibitory neurons using the patterning approach to model RTT and later transduced with neuron-specific GECI. The authors then integrated the cortical and ganglionic organoids into assembloids and performed 2-photon calcium imaging, and they observed a hyperactive and hyper-synchronized phenotype in the assembloids with mutant ganglionic organoids regardless of the genotype of cortical organoid, suggesting the role of interneurons in the network dysfunction. Future studies can transduce cortical and ganglionic organoids with different GECIs to investigate the intrinsic functional behavior of each cell type in healthy and disease states using calcium imaging.

### 3.4 Comparing functional assays

The functional assays discussed above are applicable for hiPSC models in *in vitro* NDD studies (Tables 1–4). Each method measures different aspects of neuronal development and activity, which can be utilized individually or complementary to other assays, providing flexibility in experimental design. However, some assays are more suitable for investigating disease mechanisms, while others are advantageous as drug screening platforms. In the following section, we focused on comparing the assays based on their performance in disease studies (Table 5) and drug screening (Table 6).

**TABLE 5:** Comparison of functional assays for hiPSC-based disease study.

Functional assays	Reported applications of hiPSC formats	Available plate-format	Allow long-term study	Spatial resolution	Temporal resolution	Skills required (besides cell culture)	Minimum equipment required
Patch Clamping	2D, 3D, co-culture	60mm dish with a coverslip	Possible but difficult	Highest (subcellular level)	Highest (sensitive to each change in voltage or current)	Preparation: High (prepare pipette, samples, and solution) Acquisition: high (locate cells and form appropriate contacts with the cells)	DIC microscope, patch pipets, patch clamping stations
LD-MEA	2D, 3D, co-culture	6-, 12-, 24-, 48-, 96-well	yes	Lowest (population)	High (detect each firing event from electrodes)	Preparation: None Acquisition: low (adjust parameters)	MEA plates and compatible MEA machine
HD-MEA	2D	1 or 6 wells	yes	High (single-cell, subcellular)	High (detect each firing event as well as signal propagation along axons)	Preparation: None Acquisition: low (adjust parameters)	MEA plates and compatible machine/software
Voltage imaging	2D	Common plate formats: 96 or 384 well plates	yes	High (single-cell)	High (each action potential)	Preparation: medium (load VIs or express GEVIs) Acquisition: medium (adjust focus and find FOV)	High-speed camera with fluorescence microscope
Calcium Imaging	2D, 3D, co-culture	Common plate formats: 96 or 384 well plates	yes	High (single-cell)	Low (delay in signal)	Preparation: medium (load CIs or express GECIs) Acquisition: medium (adjust focus)	Fluorescence microscope

**TABLE 6:** Applications of each functional assay for drug screening.

Functional assay as drug screening	Equipment	Example	Throughput	Number of compounds screened
Automated patch clamping	PatcherBot (contact authors)	Kolb et al., 2019	16 cells per hour	N/A
	Syncropatch 768 PE (Nanion Technology)	Vanoye et al., 2022	384 cells at the same time	Tested 39 epilepsy-associated KCNQ variations, 9480 cells total
	Qube 384 (Sophion Bioscience)	Kilfoil et al., 2021	384 wells at the same time	Tested 35 compounds on the ion channel function of hiPSC-cardiomyocytes compared to the clinical data
LD-MEA	Axion Biosystems	Bradley et al., 2018	48 wells at the same time	Tested 20 seizurogenic compounds
Voltage imaging	Firefly instrument (Quiver Bioscience)	Williams et al., 2024	Screen over 500,000 neurons plated on 96 well plates per day	Tested 29,250 compounds on ∼300 96-well plates
Calcium imaging	StemoniX	Negraes et al., 2021	384 wells	Tested 1112 compounds for neurologic research on CDD spheroids

#### 3.4.1 Disease mechanism study of NDD

Studying NDDs involves investigating the biological causes of the disorders to better understand underlying mechanisms and develop translational treatments (Benam et al., 2015). The hiPSC model provides unique opportunities to reveal the molecular and cellular aspects of NDDs in a scalable, reproducible, and human-related approach. Therefore, it is critical for the supporting functional assays to detect the changes in sub-cellular, cellular, or network activities in the hiPSC model, to distinguish between healthy and aberrant behaviors, and to validate that the model recapitulates the clinical presentation of the disorders. In addition, long-term functional studies allow for an understanding of brain development, profiling disease progression, and identifying critical time points for therapeutic intervention, which can provide translational results for drug development.

##### 3.4.1.1 Different spatial and temporal resolutions describe different aspects of neuronal activities

Patch clamping has the highest spatial and temporal resolution among the four assays. Due to the nature of the technique, patch clamping characterizes the subcellular features, such as ion channel functions, action potential waveforms, and subthreshold current changes in real-time (Table 1). Therefore, it has been the benchmark for other functional assays. It is a great tool for studying NDDs associated with channelopathy (e.g., *SCN* gene family for sodium channel; *KCN* gene family for potassium channel) (Quraishi et al., 2019; Qu et al., 2024). In addition, because of the high temporal resolution, it can be used to measure the acute drug effect on the cells a few minutes after administration (Sharma et al., 2023). However, patch clamping rarely reports network function from a population of cells due to technical constraints of the low throughput assay.

LD-MEA has the lowest spatial resolution among the assays, but higher temporal resolution than the imaging-based approaches. It reports the functional activity at each electrode and network activities of the whole well, but it does not support measuring activities from individual cells. Studies utilize LD-MEA to examine the firing patterns (e.g., burst frequency, burst duration, etc.; Table 2) as well as the firing synchronization. On the other hand, HD-MEA has high spatial resolutions that can be used for axon tracing, dendrite measurements, and signal propagation recording. In general, MEA has largely been utilized to study network activities in NDDs, such as TSC (Winden et al., 2019; Winden et al., 2023), RTT (Pradeepan et al., 2024), and *MEF2C* deficiency (Mohajeri et al., 2022; Table 2) and remains a popular method for measuring electrophysiological activities.

Optical methods (i.e., voltage and calcium imaging) have the second-highest spatial resolution with genetically encoded indicators to target specific cell types. They measure neuronal activity (e.g., amplitude, frequency, etc.) at the single-cell level and network activity (e.g., synchrony) at the population level (Marchetto et al., 2010; Saber et al., 2018; Tables 3, 4). Between the two methods, voltage imaging has a higher spatial resolution than calcium imaging because the former measures the synaptic input from the dendrite and can record subthreshold membrane potential changes. On the other hand, calcium imaging measures the output of an action potential but fails to detect non-spike-evoked calcium events (Antic et al., 2016; Zhang et al., 2023a). An additional advantage of fluorescence-based assays is that they can conveniently measure the morphological difference between the proband and wild-type cultures. Furthermore, calcium imaging also reports information about calcium homeostasis, an important factor in synaptic plasticity and network formation and dysfunction (Avazzadeh et al., 2019; Parnell et al., 2023).

In general, imaging-based assays have lower temporal resolution than electrophysiology assays because the conformational change required for fluorescent proteins to emit light introduces delays to the recording time, but voltage imaging responds to an AP event faster than calcium imaging (Figure 2E; Gonzalez M. A. et al., 2021; Zhu et al., 2021). However, newly developed voltage and calcium indicators with more rapid and sensitive kinetics are emerging, making them more desirable functional assay options (Zhang et al., 2023a; Evans et al., 2023). Although there is undoubtably room for improvement, imaging-based functional assays have contributed to the understanding of NDDs, such as RTT (Dong et al., 2018), SSADHD (Afshar-Saber et al., 2024b), and FXS (Brighi et al., 2021; Tables 3, 4).

##### 3.4.1.2 Long-term recordings allow the investigation of network development and maturation

An important part of NDD research is to understand the effects of the disorder as the brain develops, which means comparing the neuronal activity of the control and the patient cell line as the culture matures over time *in vitro*. Therefore, the functional assay should support long-term monitoring of the growth and behavior of the cell culture. Patch clamping is not suited for long-term studies. The physical process of forming a seal and injecting intracellular solutions compromises cell viability. Setting up a recording session is also time-consuming and hard to execute repeatedly, thus often limiting experimental sample size. Consequently, patch clamping is usually complemented with MEA or calcium imaging for long-term data collection. For example, Sundberg et al. (2021) utilized both patch clamping and HD-MEA to demonstrate that 16p11.2 deletion in hiPSCs-derived dopaminergic neurons have a hyperexcitable phenotype compared to controls and 16p11.2 duplication cells, which was consistent over 4 weeks.

On the other hand, MEA and imaging-based assays are both non-invasive and viable options for longitudinal studies of the hiPSC cultures. Setting up a recording session for either assay is relatively straightforward, so they can be repeated multiple times throughout the day if needed. Studies using the MEA usually last for 3 weeks (Table 2), and calcium imaging can record from over 15-week-old cultures (Fink et al., 2017). However, there are things to be cautious of when conducting long-term studies using either assay. For example, monolayer cultures often start to peel off at the edge of the wells as the cells mature due to routine media change. In MEA, the peeling around the electrodes could affect the measurement and downstream data analysis. In imaging-based assays, the effect of peeling can be mediated by choosing a different field of views in the well, but this may introduce selection bias. In addition, phototoxicity is a common issue specific to fluorescence-based methods, but it can be minimized by implementing several strategies described in other review papers (Tosheva et al., 2020; Kiepas et al., 2020).

#### 3.4.2 Drug screening platform

The purpose of drug screening *in vitro* is to identify the effective compounds that can rescue the disease phenotypes without causing neurotoxicity or compromising cellular function. As discussed in the previous sections, hiPSCs provide a promising model system for early-stage drug discovery and development. Choosing the appropriate functional assays for the hiPSC model while achieving the goal of drug screening is critical and should be: (1) suitable for high throughput screening (HTS), and (2) cost-efficient for human labor, time, and equipment. HTS, within the context of hiPSC research, mainly refers to screening a large number of drug candidates in a relatively short amount of time. Common HTS plate formats include 384, 1586, or greater number of wells on a single plate. The goal for a HTS is to identify the hit compounds and effective dose among hundreds or thousands of potential candidates at different concentrations within a week or a day. Therefore, the functional assays not only need to support a large plate format, but they also should be capable of running data acquisition and analysis at a rapid speed.

Traditional patch clamping is not feasible for drug screening due to its aforementioned shortcomings, but APC has the potential to perform as an excellent platform. Many efforts have been put into increasing the throughput of APC while decreasing human labor, such as the patcherBot_Pharma_, which is an automated robotic patch-clamping system (Kolb et al., 2019; Perszyk et al., 2021), the Qube384 fixed 384-well APC system (i.e., plates with open chambers at the bottom of each well to form seals for patch clamping (Sophion Bioscience) (Seibertz et al., 2022), or APC with multiple pipettes (Yip et al., 2024). However, each of these methods has drawbacks, and the throughput is not satisfying. The main limitation of APC, and patch clamping in general, is that it requires direct contact with single cells to obtain the data, and cell detection and pipet attachment can take up a significant amount of time. Among the attempts, Vanoye et al. (2022) successfully performed APC on 9480 cells from a 384 well plate (Nanion Technologies) to characterize the electrophysiology of different epilepsy-associated *KCNQ2* variants and tested the effect of retigabine on all the cells, which is promising for APC to work as a HTS platform. Yet, high-throughput APC requires additional or specialized equipment to achieve full automation, which can be a financial hurdle for academic research groups.

On the other hand, commercially available LD-MEA supports up to 96 wells (e.g., Axion Biosystems) on a plate due to restrictions of surface area and the number of electrodes in one well necessary for sufficient data collection. Nonetheless, each electrode serves as a recording probe, accumulating a large volume of data from one plate. In addition, LD-MEA allows simultaneous recordings from all the wells, and the recording time for one plate typically takes a relatively short amount of time. Therefore, it is possible to record multiple plates in a day to make up for the fewer wells, without purchasing separate equipment for high-content screening. For instance, Bradley et al. (2018) used a 48-well MEA plate to test 20 compounds on seizurogenic and neurotoxic effects with 15-min recordings for baseline and treatment, and they established several parameters for future screening. Groups have also turned to personally customizing MEA plates with higher throughput (Smith et al., 2020). Hence, MEA is a viable option for HTS of drug candidates.

Imaging-based assays are by far the most popular HTS platform. The methods are not limited by the physical contact with the culture as patch clamping or MEA do, so they support plates with 96, 384, or even more wells. In one study, Negraes et al. (2021) tested 1112 compounds on cortical organoids of CDD using calcium imaging with FLIPR Calcium 6 Dye (Molecular Devices) in a 384-well plate and identified four that rescued the hypersynchronous phenotype. Williams et al. (2024) utilized voltage imaging as the HTS platform on TSC2-deficient hiPSC-derived neurons and identified 434 out of 29250 small molecules that could alter the diseased phenotype. Another interesting method is to combine voltage imaging with calcium imaging. Nguyen et al. (2019) described a system where both voltage traces and calcium traces can be recorded at the same time, adding more data output and potential compounds to test. While using a regular fluorescence microscope is sufficient for conducting HTS in 2D cultures, various platforms with additional features, such as automated liquid handling (DuBreuil et al., 2021) or an ultra-widefield microscope (Werley et al., 2017), have been reported, and some are suitable for screening cortical organoids (Sirenko et al., 2019; Sakaguchi et al., 2019; Lu et al., 2023). Notably, while some high-content imaging-based platforms only support recording from one well at a time, which increases the recording time as the number of wells scales up, other platforms, such as Hamamatsu, provide simultaneous whole-plate recording at the cost of the single-cell level resolution.

## 4 Harnessing the power of machine learning for neurodevelopmental disorder research

Artificial Intelligence (AI) is a set of computer algorithms that can perform advanced tasks, such as vision, language processing, and reasoning. In recent years, AI has experienced rapid and exciting advancements, boosting revolutions in fields outside of computer science including biology and biomedicine. Machine learning (ML) is a subset of AI that is trained on collected data (i.e., learning) to produce outputs, such as classification and predictions, on a whole new dataset. If trained properly, ML can identify patterns from a large dataset with high accuracy that might not otherwise be obvious to researchers or physicians (Bejnordi et al., 2017; Hekler et al., 2019). A variety of ML methods, such as K-means clustering, logistic regression, and artificial neural networks (ANN), have been utilized in research in several fields of neuroscience, including Alzheimer’s Disease (Klöppel et al., 2008; Levakov et al., 2020), pain studies (Zhang Z. et al., 2022), and connectomic studies (Shapson-Coe et al., 2024).

**FIGURE 3:**
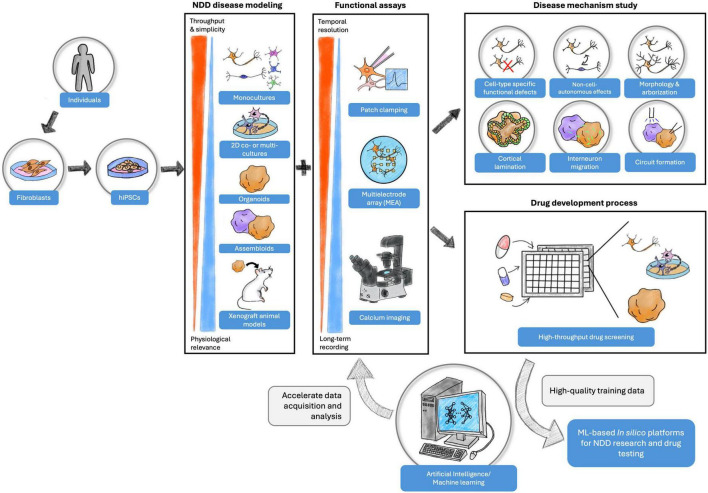
Experiment designs with the combination of hiPSC models, functional assays, and machine learning for NDD disease mechanism study and drug development process. Each hiPSC model format offers unique advantages of studying NDD *in vitro*. The functional assays provide opportunities to examine the functional properties of cell cultures at various levels and for different purposes. Finally, the high-quality data collected from the experiments can be used to build *in silico* platforms for future investigations to advance NDD research.

NDDs remain critical health problems worldwide, with at least 4.7% of the global population affected by one NDD (Francés et al., 2022). However, the current understanding of the NDDs is not sufficient for developing effective treatment or establishing thorough and objective evaluation for diagnoses. Given the recent advancements, ML has the potential to address the challenges (Figure 3). Clinically, ML can integrate and analyze different types of test data, such as fMRI, EEG, and behavioral scoring, and provide a suggestive diagnosis to assist physicians in NDD assessment and patient outcomes (Movaghar et al., 2022). The clinical applications of various ML algorithms in NDDs are discussed in other review papers (Song et al., 2022; Moreau et al., 2023). Preclinically, ML can speed up disease research and drug screening *in vitro*. As discussed previously, a significant amount of information can be extracted from experiments using hiPSC models, where ML can provide meaningful support or be trained to make predictions. In the following section, we focused on the possible ways that ML can assist functional assays and other biological studies using hiPSC models to better understand NDDs, as well as the limitations and pitfalls to avoid.

### 4.1 ML accelerates the data acquisition and analysis process

Manual patch clamping suffers from the laborious pre-recording setups and technical requirements, but various ML algorithms were developed to assist the process, such as target cell identification (Koos et al., 2021; Yip et al., 2021) and pipet correction (Gonzalez M. M. et al., 2021). The incorporation of ML makes patch clamping less time-consuming and more accessible to researchers, meanwhile enabling sequential recordings on multiple cells without extensive human operations. Automated cell detection in the APC system can increase the throughput of recording from neurons in intact brain organoids to obtain insightful information about neuronal activity and network formation from a more *in vivo*-like model. Furthermore, ML methods have been used in patch clamping data analysis to study ion channel functions (Celik et al., 2020) and synaptic signaling (Seeman et al., 2018). For example, Richter-Laskowska et al. (2021) utilized k-nearest neighbor (KNN) (i.e., a supervised classifier to group data) and autoencoder neural network (i.e., an unsupervised ANN to recreate the input data) to distinguish different cell types and cell lines based on voltage-gated and calcium-gated potassium channel activities in patch clamping data with decent accuracy. Their results demonstrated the potential applications of ML-powered analysis in identifying patterns or abnormalities of ion channel functions in patch clamping data that might otherwise be overlooked.

Additionally, ML can assist in interpreting the inter-neuronal connectivity and development measured by MEA assays. For example, Cabrera-Garcia et al. (2021) applied K-means clustering and a self-organizing map to identify characteristics of firing activities from MEA data in mouse cortical neurons at an early developmental stage. The researchers then trained three different ML models on the firing patterns to predict the mature electrical activities, and the models showed high accuracy with the MEA recording, suggesting that early firing activity could predict the development trajectory. Furthermore, ML-based spike sorting algorithms (Hilgen et al., 2017; Chaure et al., 2018) can assist in the data analysis to distinguish different cell types (Buccino et al., 2018). For example, Habibey et al. (2022) employed spike sorting algorithms in HD-MEA data to investigate the maturation of hiPSC-derived 2D neurons and the effect of GABAergic neurons on network activity. In addition, a trained ML model can be used to classify disease states based on MEA data. Matsuda et al. (2022) trained a model to predict the seizure-liability of drugs from MEA raster plots. This model classified the compounds with almost perfect accuracy, included dose effects in the output, and identified the mechanism of action, making MEA a more appealing option for high-throughput drug screening.

In terms of optical assays, retrieving single-cell level information requires labeling the cells, which is a critical yet time-consuming task and is prone to human errors and biases. Thankfully, various cell segmentation algorithms using ML, such as convolutional neural network (CNN), accelerate the image processing step and increase the efficiency of the assay (Stringer and Pachitariu, 2019; Berg et al., 2019; Pachitariu and Stringer, 2022; Zhang et al., 2023b). Several calcium imaging analysis programs have incorporated ML-based cell segmentation in their pipeline (Pachitariu et al., 2016; Kolar et al., 2021). Liu et al. (2024) generated hiPSC-derived cortical organoids of Huntington’s disease and found altered neurogenesis and corticogenesis. In the follow-up calcium imaging where they applied Mesmerize, an ML-powered calcium imaging analysis system (Kolar et al., 2021), the authors detected a decreased calcium activity amplitude and frequency in the proband organoids compared to the control group, suggesting that the observed aberrant organoid development could disrupt the neuronal network. The other challenge for data analysis in optical methods is identifying the signal peaks, and several ML algorithms have been trained to detect peaks in the recording (Sebastian et al., 2021; Zhou et al., 2023).

### 4.2 Building ML-powered *in silico* models for NDDs

Another exciting possibility about ML is building *in silico* models based on the large volume of functional data collected *in vitro*. The recent advancement of AlphaFold models, which are AI systems that can predict protein structures based on given amino acid sequences (Jumper et al., 2021; Varadi et al., 2022; Abramson et al., 2024), proves that ML can utilize complicated rules to predict a biologically plausible outcome and has been applied to NDDs (Herbst et al., 2024). Currently, most *in silico* models for NDDs are mathematics-based (El Ghaleb et al., 2021; Doorn et al., 2023), but with ML, such models can achieve more. Trujillo et al. (2019) employed a linear regression model, ElasticNet, that was trained on electroencephalogram (EEG) data from preterm infants to predict the organoid development time *in vitro* based on the MEA recording. The model prediction showed a significant and positive correlation between prenatal EEG and organoid MEA recordings, suggesting that ML algorithms can be applied to model developmental trajectories. Alternatively, the ML-based *in silico* model can be applied as a drug development tool. In another study, Trujillo et al. (2021) built an ANN based on the published data from hiPSC-derived neurons with *MeCP2* mutations to parameterize synaptic properties. The model predicted that treating synaptic defects was sufficient to rescue the decreased network activity in the *MeCP2*-mutated cells and the *in vitro* administrations of Nefiracetam and PHA543613, two drugs that increase synaptogenesis, were found to rescue the cellular activity and the MEA spike frequency in the *MeCP2*-KO neuronal cultures, which validated the model prediction.

In addition, several emerging ANN structures present possibilities for building biologically plausible, data-constrained computational simulations of population-level cellular behavior. Two examples are spiking neural network (SNN) and recurrent neural network (RNN). SNN is inspired by the brain circuit and mimics how biological neurons receive a train of spikes with frequency and inter-spike intervals, rather than discrete values, as input and output signals to other connected neurons (Yamazaki et al., 2022). SNN models based on different brain regions, including cerebellum (Vijayan and Diwakar, 2022), visual cortex (Fu et al., 2012), and basal ganglia (Girard et al., 2021), have contributed to the understanding of neural circuitry and have the potential to model neurological disorders for studying the circuit-level dysfunction. Yamaura et al. (2020) built a human-scale cerebellar SNN model with 68 billion artificial neurons based on electrical and anatomical data and simulated similar firing patterns observed in animal experiments. Although the computing time is significantly slower than biological time, it showcases the potential of constructing a model to computationally reproduce brain activity in the disease state across multiple regions.

On the other hand, RNN require time-series but not event-driven data, and the artificial neurons, or nodes, in the model receive and generate signals from each other in a connected circuit. The advantage of RNN is that the prediction is constrained by the experimental training data and can be used to study the underlying dynamics in the network that might be otherwise hard to assess (Perich and Rajan, 2020). For example, Andalman et al. (2019) trained an RNN model on single-cell level calcium imaging data from larval zebrafish and identified a potential mechanism of habenula neurons recruitment when the animals experience high level of stress. Several brain functions, such as memory (Fisher et al., 2013) and movement (Sussillo et al., 2015), have been simulated by RNN models and other new advancements, such as Current-based Decomposition (CURBD) (Perich et al., 2021), which connects multiple RNN models to simulate inter-region connections. Constructing an RNN model for NDDs using functional data of various cell populations obtained with hiPSC models could potentially reveal disease mechanisms between different cell types and brain regions, providing information for future research directions.

### 4.3 Limitations and pitfalls of ML application for NDD research

Although ML presents promising opportunities to advance the understanding of NDDs, it has limitations and pitfalls to be aware of before being utilized. First, quantity and quality of the training data are essential for an accurate and reliable model performance because the input into an ML model determines the output. The quantity of data refers to the sample size. A small sample size often results in low effect size and data overfitting, and the model often fails to detect the true effects (Rajput et al., 2023). Notably, the accuracy of model prediction is found to increase as the sample size increases (Chu et al., 2012; Cui and Gong, 2018). Rajput et al. (2023) proposed two criteria for determining the sufficient sample size based on the model prediction accuracy and effect size, but they also pointed out that the beneficial effects of increased sample size plateaued after reaching a certain number. However, the sample size needed for each ML method might vary with the complexity of the model. Infante et al. (2023) estimated that a Random Forest requires at least 150% larger sample size than other traditional regression models, and a deep learning neural network might require more than 200% than the minimum size to achieve the same performance level as statistical methods.

Nonetheless, the quality of data is equally important as the quantity. One important factor that can decrease the data quality is data bias, which includes selection bias, framing bias, and label bias (Fabbrizzi et al., 2024). For example, when using CNN for image segmentation, accurate labeling in the training data is essential for achieving the desired model performance. Precautions should also be taken when designing the experiments to include correct experimental controls and conditions to establish the baseline. In addition, data inconsistency and incompleteness also decrease the quality of data, resulting in unreliable model output. Low-quality training data often leads to decreased model performance and misleading output, so careful evaluation steps of the dataset before training have been suggested and are beneficial for studies using ML methods (Norori et al., 2021; Gong et al., 2023).

Furthermore, output explainability and interpretation are also worth noting as challenges when using ML in research. ML, especially ANN, is usually referred to as a ‘black box’ because the models produce output without an explanation on the process. Therefore, it is essential to check the model prediction, conduct cross-validations, and validate the results using *in vitro* or *in vivo* experiments when necessary. One recommendation is to use an ML-based *in silico* model as a hypothesis-generating platform and conduct biological experiments to test the idea. In addition, an ML model represents the pattern observed from the given data, so the scope of its application is limited (Williamson et al., 2019). A more precise *in silico* model of NDD would require the consideration of non-genetic factors, such as environment, nutrient, and social interactions, which are difficult to quantify and simulate.

Another important consideration is whether it is necessary to use ML in NDD studies. For example, for peak detection in calcium imaging data, besides spike detection ML algorithms, there are also mathematical methods that require less computing power and no training effort, and yet label the peaks with sufficient accuracy (Jang and Nam, 2015; Artimovich et al., 2017). Additionally, the potential financial cost for high-quality and sufficient datasets as well as computing power could outweigh the benefits of ML. Thus, one needs to determine the suitable approach by examining different methods. After all, ML is one of the many useful up-and-coming tools that can help researchers with experiments, and an informed decision based on the careful evaluation of the advantages and limitations will save time and energy to achieve accurate results.

## 5 Discussion

In this review, we provided a comprehensive overview of how functional assays and ML algorithms support and advance the study of NDD mechanisms and drug development using hiPSC-based models (Figure 3). We first compared 2D and 3D formats, and we discussed the applications of functional assays–patch clamping, MEA, and imaging-based approaches–in different hiPSC culture formats for NDD disease studies and drug screening. Finally, we explored the implementations of ML in various aspects of the NDD research process. The combination of hiPSC-based disease models, functional assays, and ML offers meaningful insights and an advanced understanding of NDDs and neuroscience (Chen et al., 2014; Russo et al., 2018; Trujillo et al., 2019; Winden et al., 2023; Afshar-Saber et al., 2024b).

Animal models have played a crucial role in enhancing our comprehension of disease mechanisms and historically, success in animal models has served as a prerequisite for advancing to clinical trials. However, their effectiveness in preclinical testing and clinical trial have shown the limitations of such models and unsuccessful clinical outcomes has raised questions about their applicability as a predictive framework for human diseases (Pankevich et al., 2014). Additionally, although human *ex vivo* brain slice efficiently recapitulates features of the human brain in health and disease, including some not observed in rodent brains or 2D cultures hiPSC cultures (Schwarz et al., 2019; Barth et al., 2021), this model present an important limitation of tissue availability (Jones et al., 2016). Regarding the hiPSC model, choosing a suitable differentiation protocol for hiPSC and understanding its limitations are essential for the experiment design and result interpretations. For example, using NGN2-overexpression method for generating 2D cultures has been reported to generate a mixed population of peripheral and CNS neurons, which can confound the experiments focused on a specific cell type (Chen et al., 2020; Lin et al., 2021). However, the addition of small-molecule patterning produces a more homogenous and mature population of cortical excitatory neurons (Nehme et al., 2018). Therefore, it is important to conduct quality control on the hiPSC-derived culture to validate the disease model. Another key question emerging in *in vitro* NDD research is whether 3D organoids can fully replace 2D models. While cerebral organoids offer notable advantages over 2D monolayer culture, including development of an in-vivo-like neural diversity and network (Jensen and Teng, 2020), they also present challenges. For example, cortical organoids are reported to show abnormal chronic stress that may affect the developmental process and cellular properties, such as functional activity and gene expression (Bhaduri et al., 2020). Additionally, generating organoids usually takes a longer time, compared to 2D culture. As Porciúncula et al. (2021) summarized, it takes at least a month for organoids to have detectable spontaneous activity and a few more months to have matured network activity, which is a substantial investment of time before data collection. Whereas in 2D cultures, for example in NGN2 neurons, spontaneous activities can be detected as early as around 10 days, with neural circuits formed after 5 weeks (Shan et al., 2024). In this case, 2D culture could provide preliminary data, while 3D organoids can be utilized for detailed network study. Moreover, as discussed previously, current functional assays are not yet fully compatible with the 3D structure to obtain the information encapsulated by the organoids. Nonetheless, ongoing research is focused on improving the long-term culturing of the brain organoid model (Giandomenico et al., 2021), cryopreservation (Xue et al., 2024), reproducibility (Glass et al., 2023; Sandoval et al., 2024) and recording technologies (Yang et al., 2024), showing promises for the extensive application of 3D organoids in NDD research in the future.

Notably, hiPSC models, including co-culture and organoids, do not capture the complexity of a biological organism, such as inclusion of the blood-brain-barrier and interactions with other organ systems, which hinders the direct application of laboratory findings to clinics. In one study (Tidball et al., 2020), three patients with SCN8A-related epilepsy were suggested to take riluzole to treating seizures based on the hiPSC results. Two patients had reduced seizure frequency during the administration but experienced various side effects, while the other did not benefit from the treatment despite the increased dosage, urging the need for more studies on variant-specific intervention as well as a better model system for understanding potential side effects. Advanced hiPSC cultures, such as assembloids (Birey et al., 2017) and gastruloids (Rossi et al., 2022), can potentially address some of the limitations. Humanized animal models (i.e., animal model with transplanted cortical organoids) could also be one solution for drug safety and efficacy tests in an *in vivo* environment, but these approaches have been accompanied by some ethical concerns. Therefore, more efforts are needed in bridging the translational gaps between hiPSC data and clinical applications.

Nonetheless, hiPSC models have provided opportunities to investigate the disease mechanisms of NDDs, especially rare and ultra-rare ones with limited patient samples or lacking established animal models (Banfi et al., 2021; Williams et al., 2022). The models also worked as an excellent platform for testing drug candidates and gene-base therapies such as ASOs or short interfering RNAs (siRNAs), thanks to the human genetic background (Chen et al., 2024). In one study, Elamin et al. (2023) administered ASO treatment to normalize *UBE3A* levels in hiPSC-derived neurons modeling chromosome 15q11-q13 duplication syndrome at an early and a late timepoint. They found that the earlier timepoint treatment rescued more phenotypes than the later administration, suggesting either an early intervention or longer treatment is necessary. Other examples of hiPSC-based drug testing include mTORC1 inhibition for TSC (Winden et al., 2019), IGF-1 treatment for RTT (Tang et al., 2016) and protein kinase inhibitor in FXS (Das Sharma et al., 2020).

This review paper also discussed four functional assays, each shedding light on the unique properties of the neuronal activities in the hiPSC culture. The methods give useful insights into the NDDs ranging from the subcellular level (e.g., ion channels) to the circuit level (e.g., network formation). We focused on comparing the assays separately in terms of their applications to NDD disease studies and drug screening, but the assays also complement each other effectively. Researchers often employ multiple assays to gain a comprehensive understanding of the research questions. Sandoval et al. (2024) recommend conducting complementary functional assays for characterizing organoids robustly, along with other suggested practices for analyzing data and reporting results. There are also a few noteworthy advancements that are expanding the hiPSC NDD field, although not addressed in this review. Optogenetics (Emiliani et al., 2022) is a tool often coupled with image-based recordings or electrophysiology, which enables selective, non-invasive stimulation of neurons via light-gated ion channels. Furthermore, non-functional assays, such as omics studies (Burke et al., 2020; Brooks et al., 2022) and Cell Painting (Bray et al., 2016), provide additional information on the cellular and molecular profiles of hiPSC-derived disease models.

In recent years, ML has gained prominence and impacted multiple areas of NDD research, particularly in data acquisition and analysis. Besides working with functional assays, ML methods can benefit multi-omics studies, which generate extensive data from hiPSC samples. ML can also enhance data analysis, integrate diverse omics datasets, and potentially identify biomarkers (Costello and Martin, 2018; Reel et al., 2021; Feldner-Busztin et al., 2023), shedding light on the developmental trajectory or dysregulated gene expression associated with the disorders (Zhu et al., 2023; Tian et al., 2024). Importantly, we discussed the limitations of using ML methods, especially neural networks, due to the requirement on high-quality data, limited applications, and potential financial costs. Nonetheless, as more studies reveal distinct mechanisms of each NDD using hiPSC models, a centralized and organized data collection platform for each NDD will help train accurate ML models as well as support scientists in sharing, reviewing, understanding, and planning for future research on the disorders (Winden et al., 2019; Birey et al., 2022; Sharma et al., 2023; Afshar-Saber et al., 2024b). The Blue Brain Project by École Polytechnique Fédérale de Lausanne, Allen Brain Atlas, and the NIH-funded NeuroLINCS project for neurodegenerative disorders are examples of how such a platform can benefit the broad neuroscience and neurodevelopment community by increasing scientific communication and transparency.

## References

[B1] Abramson J. Adler J. Dunger J. Evans R. Green T. Pritzel A. (2024). Accurate structure prediction of biomolecular interactions with AlphaFold 3. *Nature* 630 493–500. 10.1038/S41586-024-07487-W 38718835 PMC11168924

[B2] Abud E. M. Ramirez R. N. Martinez E. S. Healy L. M. Nguyen C. H. H. Newman S. A. (2017). iPSC-derived human microglia-like cells to study neurological diseases. *Neuron* 94 278–293.e9. 10.1016/J.NEURON.2017.03.042 28426964 PMC5482419

[B3] Afshar-Saber W. Chen C. Teaney N. A. Kim K. Yang Z. Gasparoli F. M. (2024a). Generation and characterization of six human induced pluripotent stem cell lines (hiPSCs) from three individuals with SSADH Deficiency and CRISPR-corrected isogenic controls. *Stem Cell Res.* 77:103424. 10.1016/J.SCR.2024.103424 38677032 PMC11178435

[B4] Afshar-Saber W. Teaney N. A. Winden K. D. Jumo H. Shi X. McGinty G. (2024b). ALDH5A1-deficient iPSC-derived excitatory and inhibitory neurons display cell type specific alterations’. *Neurobiol. Dis.* 190:106386. 10.1016/J.NBD.2023.106386 38110041 PMC10843729

[B5] Ahtiainen A. Genocchi B. Tanskanen J. M. A. Barros M. T. Hyttinen J. A. K. Lenk K. (2021). ‘Astrocytes exhibit a protective role in neuronal firing patterns under chemically induced seizures in neuron–astrocyte co-cultures’. *Int. J. Mol. Sci.* 22:12770. 10.3390/IJMS222312770/S1 PMC8657549 34884577

[B6] Ali F. Kwan A. C. (2020). ‘Interpreting in vivo calcium signals from neuronal cell bodies, axons, and dendrites: a review’. *Neurophotonics* 7:011402. 10.1117/1.NPH.7.1.011402 31372367 PMC6664352

[B7] Alich T. C. Röderer P. Szalontai B. Golcuk K. Tariq S. Peitz M. (2023). Bringing to light the physiological and pathological firing patterns of human induced pluripotent stem cell-derived neurons using optical recordings’. *Front. Cell. Neurosci.* 16:1039957. 10.3389/FNCEL.2022.1039957 36733665 PMC9887032

[B8] Andalman A. S. Burns V. M. Lovett-Barron M. Broxton M. Poole B. Yang S. J. (2019). Neuronal dynamics regulating brain and behavioral state transitions’. *Cell* 177 970–985.e20. 10.1016/J.CELL.2019.02.037 31031000 PMC6726130

[B9] Andersen J. Revah O. Miura Y. Thom N. Amin N. D. Kelley K. W. (2020). Generation of functional human 3D cortico-motor assembloids’. *Cell* 183 1913–1929.e26. 10.1016/J.CELL.2020.11.017 33333020 PMC8711252

[B10] Anderson N. C. Chen P. F. Meganathan K. Afshar Saber W. Petersen A. J. Bhattacharyya A. (2021). ‘Balancing serendipity and reproducibility: pluripotent stem cells as experimental systems for intellectual and developmental disorders’. *Stem Cell Rep.* 16:1446. 10.1016/J.STEMCR.2021.03.025 33861989 PMC8190574

[B11] Antic S. D. Empson R. M. Knöpfel T. (2016). ‘Voltage imaging to understand connections and functions of neuronal circuits’. *J. Neurophysiol.* 116 135–152. 10.1152/JN.00226.2016 27075539 PMC4961759

[B12] Antolini G. Colizzi M. (2023). Where do neurodevelopmental disorders go? Casting the eye away from childhood towards adulthood. *Healthcare (Switzerland)* 11:1015. 10.3390/HEALTHCARE11071015 37046942 PMC10094062

[B13] Artimovich E. Jackson R. K. Kilander M. B. C. Lin Y. C. Nestor M. W. (2017). PeakCaller: an automated graphical interface for the quantification of intracellular calcium obtained by high-content screening. *BMC Neurosci.* 18:72. 10.1186/S12868-017-0391-Y 29037171 PMC5644055

[B14] Aseyev N. Ivanova V. Balaban P. Nikitin E. (2023). Current practice in using voltage imaging to record fast neuronal activity: successful examples from invertebrate to mammalian studies. *Biosensors* 13:648. 10.3390/BIOS13060648 37367013 PMC10296598

[B15] Avazzadeh S. McDonagh K. Reilly J. Wang Y. Boomkamp S. D. McInerney V. (2019). Increased Ca2+ signaling in NRXN1α+/- neurons derived from ASD induced pluripotent stem cells. *Mol. Autism* 10:52. 10.1186/S13229-019-0303-3 31893021 PMC6937972

[B16] Avazzadeh S. Quinlan L. R. Reilly J. McDonagh K. Jalali A. Wang Y. (2021). NRXN1α+/- is associated with increased excitability in ASD iPSC-derived neurons. *BMC Neurosci.* 22:56. 10.1186/S12868-021-00661-0 34525970 PMC8442436

[B17] Banfi F. Rubio A. Zaghi M. Massimino L. Fagnocchi G. Bellini E. (2021). ‘SETBP1 accumulation induces P53 inhibition and genotoxic stress in neural progenitors underlying neurodegeneration in Schinzel-Giedion syndrome’. *Nat. Commun.* 12:4050. 10.1038/S41467-021-24391-3 34193871 PMC8245514

[B18] Barth M. Bacioglu M. Schwarz N. Novotny R. Brandes J. Welzer M. (2021). ‘Microglial inclusions and neurofilament light chain release follow neuronal α-synuclein lesions in long-term brain slice cultures’. *Mol. Neurodegen.* 16:54. 10.1186/S13024-021-00471-2/FIGURES/5 34380535 PMC8356412

[B19] Beck C. Gong Y. (2019). A high-speed, bright, red fluorescent voltage sensor to detect neural activity. *Sci. Rep.* 9:15878. 10.1038/S41598-019-52370-8 31685893 PMC6828731

[B20] Bejnordi B. E. Veta M. Van Diest P. J. Van Ginneken B. Karssemeijer N. Litjens G. (2017). ‘Diagnostic assessment of deep learning algorithms for detection of lymph node metastases in women with breast cancer’. *JAMA* 318 2199–2210. 10.1001/JAMA.2017.14585 29234806 PMC5820737

[B21] Belinsky G. S. Rich M. T. Sirois C. L. Short S. M. Pedrosa E. Lachman H. M. (2014). ‘Patch-clamp recordings and calcium imaging followed by single-cell PCR reveal the developmental profile of 13 genes in iPSC-derived human neurons’. *Stem Cell Res.* 12 101–118. 10.1016/J.SCR.2013.09.014 24157591 PMC3947234

[B22] Benam K. H. Dauth S. Hassell B. Herland A. Jain A. Jang K. J. (2015). ‘Engineered in vitro disease models’. *Annu. Rev. Pathol.* 10 195–262. 10.1146/ANNUREV-PATHOL-012414-040418 25621660

[B23] Berg S. Kutra D. Kroeger T. Straehle C. N. Kausler B. X. Haubold C. (2019). ‘ilastik: interactive machine learning for (bio)image analysis’. *Nat. Methods* 16 1226–1232. 10.1038/S41592-019-0582-9 31570887

[B24] Bhaduri A. Andrews M. G. Mancia Leon W. Jung D. Shin D. Allen D. (2020). ‘Cell stress in cortical organoids impairs molecular subtype specification’. *Nature* 578 142–148. 10.1038/S41586-020-1962-0 31996853 PMC7433012

[B25] Birey F. Andersen J. Makinson C. D. Islam S. Wei W. Huber N. (2017). ‘Assembly of functionally integrated human forebrain spheroids.’. *Nature* 545 54–59. 10.1038/NATURE22330 28445465 PMC5805137

[B26] Birey F. Li M. Y. Gordon A. Thete M. V. Valencia A. M. Revah O. (2022). Dissecting the molecular basis of human interneuron migration in forebrain assembloids from Timothy syndrome. *Cell Stem Cell* 29 248–264.e7. 10.1016/J.STEM.2021.11.011 34990580 PMC13492730

[B27] Bogdańska-Chomczyk E. Równiak M. Huang A. C. W. Kozłowska A. (2024). Parvalbumin interneuron deficiency in the prefrontal and motor cortices of spontaneously hypertensive rats: an attention-deficit hyperactivity disorder animal model insight. *Front. Psychiatry* 15:1359237. 10.3389/FPSYT.2024.1359237 38600979 PMC11005678

[B28] Bonti E. Zerva I. K. Koundourou C. Sofologi M. (2024). The high rates of comorbidity among neurodevelopmental disorders: reconsidering the clinical utility of distinct diagnostic categories. *J. Pers. Med.* 14:300. 10.3390/JPM14030300 38541042 PMC10971064

[B29] Bradley J. A. Luithardt H. H. Metea M. R. Strock C. J. (2018). ‘In vitro screening for seizure liability using microelectrode array technology’. *Toxicol. Sci.* 163 240–253. 10.1093/TOXSCI/KFY029 29432603

[B30] Braubach O. Cohen L. B. Choi Y. (2015). ‘Historical overview and general methods of membrane potential imaging’. *Adv. Exp. Med. Biol.* 859 3–26. 10.1007/978-3-319-17641-3_1 26238047

[B31] Bray M. A. Singh S. Han H. Davis C. T. Borgeson B. Hartland C. (2016). ‘Cell Painting, a high-content image-based assay for morphological profiling using multiplexed fluorescent dyes’. *Nat. protoc.* 11 1757–1774. 10.1038/NPROT.2016.105 27560178 PMC5223290

[B32] Brighi C. Salaris F. Soloperto A. Cordella F. Ghirga S. de Turris V. (2021). Novel fragile X syndrome 2D and 3D brain models based on human isogenic FMRP-KO iPSCs. *Cell Death Dis.* 12:498. 10.1038/S41419-021-03776-8 33993189 PMC8124071

[B33] Bromfield E. B. Cavazos J. E. Sirven J. I. (2006). “An Introduction to Epilepsy [Internet],” in *An Introduction to Epilepsy [Internet]*, eds Bromfield E. B. Cavazos J. E. Sirven J. I. (West Hartford, CT: American Epilepsy Society). 20821849

[B34] Brooks I. R. Garrone C. M. Kerins C. Kiar C. S. Syntaka S. Xu J. Z. (2022). ‘Functional genomics and the future of iPSCs in disease modeling’. *Stem Cell Rep.* 17 1033–1047. 10.1016/J.STEMCR.2022.03.019 35487213 PMC9133703

[B35] Bu Q. Dai Y. Zhang H. Li M. Liu H. Huang Y. (2023). Neurodevelopmental defects in human cortical organoids with N-acetylneuraminic acid synthase mutation. *Sci. Adv.* 9:eadf2772. 10.1126/SCIADV.ADF2772 38000033 PMC10672180

[B36] Buccino A. P. Kordovan M. Ness T. V. Merkt B. Häfliger P. D. Fyhn M. (2018). ‘Combining biophysical modeling and deep learning for multielectrode array neuron localization and classification’. *J. Neurophysiol.* 120 1212–1232. 10.1152/JN.00210.2018 29847231

[B37] Burke E. E. Chenoweth J. G. Shin J. H. Collado-Torres L. Kim S. K. Micali N. (2020). Dissecting transcriptomic signatures of neuronal differentiation and maturation using iPSCs. *Nat. Commun.* 11:462. 10.1038/S41467-019-14266-Z 31974374 PMC6978526

[B38] Bury L. A. D. Fu S. Wynshaw-Boris A. (2024). ‘Neuronal lineage tracing from progenitors in human cortical organoids reveals mechanisms of neuronal production, diversity, and disease’. *Cell Rep.* 43:114862. 10.1016/j.celrep.2024.114862 39395167

[B39] Cabrera-Garcia D. Warm D. de la Fuente P. Fernández-Sánchez M. T. Novelli A. Villanueva-Balsera J. M. (2021). Early prediction of developing spontaneous activity in cultured neuronal networks. *Sci. Rep.* 11 10.1038/S41598-021-99538-9 34650146 PMC8516856

[B40] Cao S. Y. Hu Y. Chen C. Yuan F. Xu M. Li Q. (2017). Enhanced derivation of human pluripotent stem cell-derived cortical glutamatergic neurons by a small molecule. *Sci. Rep.* 7:3282. 10.1038/S41598-017-03519-W 28607372 PMC5468244

[B41] Celik N. O’Brien F. Brennan S. Rainbow R. D. Dart C. Zheng Y. (2020). Deep-Channel uses deep neural networks to detect single-molecule events from patch-clamp data. *Commun. Biol.* 3:3. 10.1038/S42003-019-0729-3 31925311 PMC6946689

[B42] Chambers S. M. Fasano C. A. Papapetrou E. P. Tomishima M. Sadelain M. Studer L. (2009). ‘Highly efficient neural conversion of human ES and iPS cells by dual inhibition of SMAD signaling’. *Nat. Biotechnol.* 27 275–280. 10.1038/NBT.1529 19252484 PMC2756723

[B43] Chandrasekaran A. Avci H. X. Ochalek A. Rösingh L. N. Molnár K. László L. (2017). ‘Comparison of 2D and 3D neural induction methods for the generation of neural progenitor cells from human induced pluripotent stem cells’. *Stem Cell Res.* 25 139–151. 10.1016/J.SCR.2017.10.010 29128818

[B44] Chaure F. J. Rey H. G. Quian Quiroga R. (2018). ‘A novel and fully automatic spike-sorting implementation with variable number of features’. *J. Neurophysiol.* 120 1859–1871. 10.1152/JN.00339.2018 29995603 PMC6230803

[B45] Chen C. Jiang P. Xue H. Peterson S. E. Tran H. T. McCann A. E. (2014). Role of astroglia in Down’s syndrome revealed by patient-derived human-induced pluripotent stem cells. *Nat. Commun.* 5:4430. 10.1038/NCOMMS5430 25034944 PMC4109022

[B46] Chen M. Maimaitili M. Habekost M. Gill K. P. Mermet-Joret N. Nabavi S. (2020). Rapid generation of regionally specified CNS neurons by sequential patterning and conversion of human induced pluripotent stem cells. *Stem Cell Res.* 48:101945. 10.1016/J.SCR.2020.101945 32791483

[B47] Chen T. W. Wardill T. J. Sun Y. Pulver S. R. Renninger S. L. Baohan A. (2013). ‘Ultrasensitive fluorescent proteins for imaging neuronal activity’. *Nature* 499 295–300. 10.1038/NATURE12354 23868258 PMC3777791

[B48] Chen X. Birey F. Li M. Y. Revah O. Levy R. Thete M. V. (2024). Antisense oligonucleotide therapeutic approach for Timothy syndrome. *Nature* 628 818–825. 10.1038/s41586-024-07310-6 38658687 PMC11043036

[B49] Chu C. Hsu A. L. Chou K. H. Bandettini P. Lin C. P. (2012). ‘Does feature selection improve classification accuracy? Impact of sample size and feature selection on classification using anatomical magnetic resonance images. *Neuroimage* 60 59–70. 10.1016/J.NEUROIMAGE.2011.11.066 22166797

[B50] Cohen L. B. Salzberg B. M. Davila H. V. Ross W. N. Landowne D. Waggoner A. S. (1974). ‘Changes in axon fluorescence during activity: molecular probes of membrane potential’. *J. Membr. Biol.* 19 1–36. 10.1007/BF01869968 4431037

[B51] Cortese S. Solmi M. Michelini G. Bellato A. Blanner C. Canozzi A. (2023). ‘Candidate diagnostic biomarkers for neurodevelopmental disorders in children and adolescents: a systematic review’. *World Psychiatry* 22 129–149. 10.1002/WPS.21037 36640395 PMC9840506

[B52] Costello Z. Martin H. G. (2018). A machine learning approach to predict metabolic pathway dynamics from time-series multiomics data. *NPJ Syst. Biol. Applic.* 4:19. 10.1038/S41540-018-0054-3 29872542 PMC5974308

[B53] Cui Z. Gong G. (2018). ‘The effect of machine learning regression algorithms and sample size on individualized behavioral prediction with functional connectivity features’. *Neuroimage* 178 622–637. 10.1016/J.NEUROIMAGE.2018.06.001 29870817

[B54] Culotta L. Penzes P. (2020). Exploring the mechanisms underlying excitation/inhibition imbalance in human iPSC-derived models of ASD. *Mol. Autism* 11:32. 10.1186/S13229-020-00339-0 32393347 PMC7216514

[B55] Damianidou E. Mouratidou L. Kyrousi C. (2022). Research models of neurodevelopmental disorders: the right model in the right place. *Front. Neurosci.* 16:1031075. 10.3389/FNINS.2022.1031075 36340790 PMC9630472

[B56] Das Sharma S. Pal R. Reddy B. K. Selvaraj B. T. Raj N. Samaga K. K. (2020). Cortical neurons derived from human pluripotent stem cells lacking FMRP display altered spontaneous firing patterns. *Mol. Autism* 11:52. 10.1186/S13229-020-00351-4 32560741 PMC7304215

[B57] de Melo Reis R. A. Freitas H. R. de Mello F. G. (2020). Cell calcium imaging as a reliable method to study neuron-glial circuits. *Front. Neurosci.* 14:569361. 10.3389/FNINS.2020.569361 33122991 PMC7566175

[B58] Dindot S. V. Christian S. Murphy W. J. Berent A. Panagoulias J. Schlafer A. (2023). An ASO therapy for Angelman syndrome that targets an evolutionarily conserved region at the start of the UBE3A-AS transcript. *Sci. Transl. Med.* 15:eabf4077. 10.1126/SCITRANSLMED.ABF4077 36947593

[B59] Dolan M. J. Therrien M. Jereb S. Kamath T. Gazestani V. Atkeson T. (2023). ‘Exposure of iPSC-derived human microglia to brain substrates enables the generation and manipulation of diverse transcriptional states in vitro’. *Nat. Immunol.* 24 1382–1390. 10.1038/S41590-023-01558-2 37500887 PMC10382323

[B60] Dong Q. Liu Q. Li R. Wang A. Bu Q. Wang K. H. (2018). Mechanism and consequence of abnormal calcium homeostasis in Rett syndrome astrocytes. *eLife* 7:e33417. 10.7554/ELIFE.33417 29595472 PMC5902163

[B61] Doorn N. van Hugte E. J. H. Ciptasari U. Mordelt A. Meijer H. G. E. Schubert D. (2023). ‘An in silico and in vitro human neuronal network model reveals cellular mechanisms beyond NaV1.1 underlying Dravet syndrome’. *Stem Cell Rep.* 18 1686–1700. 10.1016/J.STEMCR.2023.06.003 37419110 PMC10444571

[B62] DuBreuil D. M. Chiang B. M. Zhu K. Lai X. Flynn P. Sapir Y. (2021). A high-content platform for physiological profiling and unbiased classification of individual neurons. *Cell Rep. Methods* 1:100004. 10.1016/J.CRMETH.2021.100004 34318289 PMC8312640

[B63] Dunn D. W. Austin J. K. Harezlak J. Ambrosius W. T. (2003). ‘ADHD and epilepsy in childhood’. *Dev. Med. Child Neurol.* 45 50–54. 10.1111/J.1469-8749.2003.TB00859.X 12549755

[B64] Ehninger D. Han S. Shilyansky C. Zhou Y. Li W. Kwiatkowski D. J. (2008). ‘Reversal of learning deficits in a Tsc2+/- mouse model of tuberous sclerosis’. *Nat. Med.* 14 843–848. 10.1038/NM1788 18568033 PMC2664098

[B65] Ehrlich M. Mozafari S. Glatza M. Starost L. Velychko S. Hallmann A. L. (2017). ‘Rapid and efficient generation of oligodendrocytes from human induced pluripotent stem cells using transcription factors’. *Proc. Natl Acad. Sci. U.S.A.* 114 E2243–E2252. 10.1073/PNAS.1614412114 28246330 PMC5358375

[B66] Eichmüller O. L. Corsini N. S. Vértesy Á Morassut I. Scholl T. Gruber V. E. (2022). Amplification of human interneuron progenitors promotes brain tumors and neurological defects. *Science (New York, N.Y.)* 375:eabf5546. 10.1126/SCIENCE.ABF5546 35084981 PMC7613689

[B67] El Ghaleb Y. Schneeberger P. E. Fernández-Quintero M. L. Geisler S. M. Pelizzari S. Polstra A. M. (2021). ‘CACNA1I gain-of-function mutations differentially affect channel gating and cause neurodevelopmental disorders’. *Brain* 144 2092–2106. 10.1093/BRAIN/AWAB101 33704440 PMC8422349

[B68] Elamin M. Lemtiri-Chlieh F. Robinson T. M. Levine E. S. (2023). ‘Dysfunctional sodium channel kinetics as a novel epilepsy mechanism in chromosome 15q11-q13 duplication syndrome’. *Epilepsia* 64 2515–2527. 10.1111/EPI.17687 37329181 PMC10529833

[B69] Emiliani V. Entcheva E. Hedrich R. Hegemann P. Konrad K. R. Lüscher C. (2022). Optogenetics for light control of biological systems. *Nat. Rev. Methods Prim.* 2:55. 10.1038/S43586-022-00136-4 37933248 PMC10627578

[B70] Eroglu C. Barres B. A. (2010). ‘Regulation of synaptic connectivity by glia’. *Nature* 468 223–231. 10.1038/NATURE09612 21068831 PMC4431554

[B71] Evans S. W. Shi D. Q. Chavarha M. Plitt M. H. Taxidis J. Madruga B. (2023). ‘A positively tuned voltage indicator for extended electrical recordings in the brain’. *Nat. Methods* 20 1104–1113. 10.1038/S41592-023-01913-Z 37429962 PMC10627146

[B72] Fabbrizzi S. Zhao X. Krasanakis E. Papadopoulos S. Ntoutsi E. (2024). ‘Studying bias in visual features through the lens of optimal transport’. *Data Mining Knowl. Discov.* 38 281–312. 10.1007/S10618-023-00972-2/FIGURES/10

[B73] Feldner-Busztin D. Nisantzis P. F. Edmunds S. J. Boza G. Racimo F. Gopalakrishnan S. (2023). Dealing with dimensionality: the application of machine learning to multi-omics data. *Bioinformatics (Oxford, England)* 39:btad021. 10.1093/BIOINFORMATICS/BTAD021 36637211 PMC9907220

[B74] Fink J. J. Robinson T. M. Germain N. D. Sirois C. L. Bolduc K. A. Ward A. J. (2017). Disrupted neuronal maturation in Angelman syndrome-derived induced pluripotent stem cells. *Nat. Commun.* 8:15038. 10.1038/NCOMMS15038 28436452 PMC5413969

[B75] Fisher D. Olasagasti I. Tank D. W. Aksay E. R. F. Goldman M. S. (2013). ‘A modeling framework for deriving the structural and functional architecture of a short-term memory microcircuit’. *Neuron* 79 987–1000. 10.1016/J.NEURON.2013.06.041 24012010 PMC3768012

[B76] Francés L. Quintero J. Fernández A. Ruiz A. Caules J. Fillon G. (2022). Current state of knowledge on the prevalence of neurodevelopmental disorders in childhood according to the DSM-5: a systematic review in accordance with the PRISMA criteria. *Child Adolesc. Psychiatry Ment. Health* 16:27. 10.1186/S13034-022-00462-1 35361232 PMC8973738

[B77] Franz D. Olsen H. L. Klink O. Gimsa J. (2017). Automated and manual patch clamp data of human induced pluripotent stem cell-derived dopaminergic neurons. *Sci. Data* 4:170056. 10.1038/SDATA.2017.56 28440808 PMC5404656

[B78] Frega M. Linda K. Keller J. M. Gümüş-Akay G. Mossink B. van Rhijn J. R. (2019). Neuronal network dysfunction in a model for Kleefstra syndrome mediated by enhanced NMDAR signaling. *Nat. Commun.* 10:4928. 10.1038/S41467-019-12947-3 31666522 PMC6821803

[B79] Fu S. Y. Yang G. S. Kuai X. K. (2012). A spiking neural network based cortex-like mechanism and application to facial expression recognition. *Comput. Intellig. Neurosci.* 2012:946589. 10.1155/2012/946589 23193391 PMC3501821

[B80] Galiakberova A. A. Surin A. M. Bakaeva Z. V. Sharipov R. R. Zhang D. Dorovskoy D. A. (2022). ‘IPSC-derived human neurons with GCaMP6s expression allow in vitro study of neurophysiological responses to neurochemicals’. *Neurochem. Res.* 47 952–966. 10.1007/S11064-021-03497-6 34855047 PMC8891101

[B81] Gao J. Liao C. Liu S. Xia T. Jiang G. (2021). Nanotechnology: new opportunities for the development of patch-clamps. *J. Nanobiotechnol.* 19:97. 10.1186/S12951-021-00841-4 33794903 PMC8017657

[B82] Gao R. Penzes P. (2015). ‘Common mechanisms of excitatory and inhibitory imbalance in schizophrenia and autism spectrum disorders’. *Curr. Mol. Med.* 15 146–167. 10.2174/1566524015666150303003028 25732149 PMC4721588

[B83] Gasterstädt I. Jack A. Stahlhut T. Rennau L. M. Gonda S. Wahle P. (2020). Genetically encoded calcium indicators can impair dendrite growth of cortical neurons. *Front. Cell. Neurosci.* 14:570596. 10.3389/FNCEL.2020.570596 33192315 PMC7606991

[B84] Giandomenico S. L. Sutcliffe M. Lancaster M. A. (2021). ‘Generation and long-term culture of advanced cerebral organoids for studying later stages of neural development’. *Nat. Protoc.* 16 579–602. 10.1038/S41596-020-00433-W 33328611 PMC7611064

[B85] Gidziela A. Ahmadzadeh Y. I. Michelini G. Allegrini A. G. Agnew-Blais J. Lau L. Y. (2023). ‘A meta-analysis of genetic effects associated with neurodevelopmental disorders and co-occurring conditions’. *Nat. Hum. Behav.* 7 642–656. 10.1038/S41562-023-01530-Y 36806400 PMC10129867

[B86] Giorgi C. Lombardozzi G. Ammannito F. Scenna M. S. Maceroni E. Quintiliani M. (2024). Brain organoids: a game-changer for drug testing. *Pharmaceutics* 16:443. 10.3390/PHARMACEUTICS16040443 38675104 PMC11054008

[B87] Girard B. Lienard J. Gutierrez C. E. Delord B. Doya K. (2021). ‘A biologically constrained spiking neural network model of the primate basal ganglia with overlapping pathways exhibits action selection’. *Eur. J. Neurosci.* 53 2254–2277. 10.1111/EJN.14869 32564449 PMC8246891

[B88] Glass M. R. Waxman E. A. Yamashita S. Lafferty M. Beltran A. Farah T. (2023). Cross-site reproducibility of human cortical organoids reveals consistent cell type composition and architecture. *bioRxiv [Preprint]* 10.1101/2023.07.28.550873 39178845 PMC11411306

[B89] Goldstein S. Schwebach A. J. (2004). ‘The comorbidity of pervasive developmental disorder and attention deficit hyperactivity disorder: results of a retrospective chart review’. *J. Autism Dev. Disord.* 34 329–339. 10.1023/B:JADD.0000029554.46570.68 15264500

[B90] Gomes A. R. Fernandes T. G. Vaz S. H. Silva T. P. Bekman E. P. Xapelli S. (2020). Modeling rett syndrome with human patient-specific forebrain organoids. *Front. Cell Dev. Biol.* 8:610427. 10.3389/FCELL.2020.610427/FULL PMC7758289 33363173

[B91] Gómez-Robles A. Nicolaou C. Smaers J. B. Sherwood C. C. (2024). ‘The evolution of human altriciality and brain development in comparative context’. *Nat. Ecol. Evol.* 8 133–146. 10.1038/S41559-023-02253-Z 38049480 PMC10781642

[B92] Gong Y. Liu G. Xue Y. Li R. Meng L. (2023). ‘A survey on dataset quality in machine learning’. *Inform. Softw. Technol.* 162:107268. 10.1016/J.INFSOF.2023.107268

[B93] Gonzalez M. A. Walker A. S. Cao K. J. Lazzari-Dean J. R. Settineri N. S. Kong E. J. (2021). ‘Voltage imaging with a NIR-absorbing phosphine oxide rhodamine voltage reporter’. *J. Am. Chem. Soc.* 143 2304–2314. 10.1021/JACS.0C11382 33501825 PMC7986050

[B94] Gonzalez M. M. Lewallen C. F. Yip M. C. Forest C. R. (2021). Machine learning-based pipette positional correction for automatic patch clamp in vitro. *eNeuro* 8 ENEURO.0051-21.2021. 10.1523/ENEURO.0051-21.2021 34312222 PMC8318343

[B95] Goorden S. M. I. Van Woerden G. M. Van Der Weerd L. Cheadle J. P. Elgersma Y. (2007). ‘Cognitive deficits in Tsc1+/-mice in the absence of cerebral lesions and seizures’. *Ann. Neurol.* 62 648–655. 10.1002/ANA.21317 18067135

[B96] Gordon A. Yoon S. J. Tran S. S. Makinson C. D. Park J. Y. Andersen J. (2021). Long-term maturation of human cortical organoids matches key early postnatal transitions. *Nat. Neurosci.* 24 331–342. 10.1038/S41593-021-00802-Y 33619405 PMC8109149

[B97] Graef J. D. Wu H. Ng C. Sun C. Villegas V. Qadir D. (2020). ‘Partial FMRP expression is sufficient to normalize neuronal hyperactivity in Fragile X neurons’. *Eur. J. Neurosci.* 51:2143. 10.1111/EJN.14660 31880363 PMC7318714

[B98] Gu F. Hazra A. Aulakh A. Žiburkus J. (2014). ‘Purinergic control of hippocampal circuit hyperexcitability in Dravet syndrome’. *Epilepsia* 55 245–255. 10.1111/EPI.12487 24417577

[B99] Habibey R. Striebel J. Schmieder F. Czarske J. Busskamp V. (2022). Long-term morphological and functional dynamics of human stem cell-derived neuronal networks on high-density micro-electrode arrays. *Front. Neurosci.* 16:951964. 10.3389/FNINS.2022.951964 36267241 PMC9578684

[B100] Halvorsen M. Mathiassen B. Myrbakk E. Brøndbo P. H. Sætrum A. Steinsvik O. O. (2019). ‘Neurodevelopmental correlates of behavioural and emotional problems in a neuropaediatric sample’. *Res. Dev. Disabil.* 85 217–228. 10.1016/J.RIDD.2018.11.005 30580152

[B101] Hechtman L. Swanson J. M. Sibley M. H. Stehli A. Lakes K. D. Owens E. B. (2016). Functional adult outcomes 16 years after childhood diagnosis of attention-deficit/hyperactivity disorder: MTA results. *J. Am. Acad. Child Adolesc. Psychiatry* 55 945–952.e2. 10.1016/J.JAAC.2016.07.774 27806862 PMC5113724

[B102] Hedegaard A. Monzón-Sandoval J. Newey S. E. Whiteley E. S. Webber C. Akerman C. J. (2020). ‘Pro-maturational effects of human iPSC-derived cortical astrocytes upon iPSC-derived cortical neurons’. *Stem Cell Rep.* 15 38–51. 10.1016/J.STEMCR.2020.05.003 32502466 PMC7363746

[B103] Hekler A. Utikal J. S. Enk A. H. Solass W. Schmitt M. Klode J. (2019). ‘Deep learning outperformed 11 pathologists in the classification of histopathological melanoma images’. *Eur. J. Cancer (Oxford, England’: 1990)* 118 91–96. 10.1016/J.EJCA.2019.06.012 31325876

[B104] Henderson M. J. Baldwin H. A. Werley C. A. Boccardo S. Whitaker L. R. Yan X. (2015). A low affinity GCaMP3 Variant (GCaMPer) for imaging the endoplasmic reticulum calcium store. *PLoS One* 10:e0139273. 10.1371/JOURNAL.PONE.0139273 26451944 PMC4599735

[B105] Herbst C. Bothe V. Wegler M. Axer-Schaefer S. Audebert-Bellanger S. Gecz J. (2024). ‘Heterozygous loss-of-function variants in DOCK4 cause neurodevelopmental delay and microcephaly’. *Hum. Genet.* 143 455–469. 10.1007/S00439-024-02655-4 38526744 PMC11043173

[B106] Hilgen G. Sorbaro M. Pirmoradian S. Muthmann J. O. Kepiro I. E. Ullo S. (2017). ‘Unsupervised spike sorting for large-scale, high-density multielectrode arrays. *Cell Rep.* 18 2521–2532. 10.1016/J.CELREP.2017.02.038 28273464

[B107] Hinz L. Hoekstra S. D. Watanabe K. Posthuma D. Heine V. M. (2019). ‘Generation of isogenic controls for in vitro disease modelling of X-chromosomal disorders’. *Stem Cell Rev. Rep.* 15 276–285. 10.1007/S12015-018-9851-8 30421281 PMC6441401

[B108] Hisatsune C. Shimada T. Miyamoto A. Lee A. Yamagata K. (2021). ‘Tuberous Sclerosis Complex (TSC) inactivation increases neuronal network activity by enhancing Ca2+ Influx via L-Type Ca2+ channels’. *J. Neurosci.* 41 8134–8149. 10.1523/JNEUROSCI.1930-20.2021 34417327 PMC8482857

[B109] Hochbaum D. R. Zhao Y. Farhi S. L. Klapoetke N. Werley C. A. Kapoor V. (2014). ‘All-optical electrophysiology in mammalian neurons using engineered microbial rhodopsins’. *Nat. Methods* 11 825–833. 10.1038/NMETH.3000 24952910 PMC4117813

[B110] Hoppe M. Habib A. Desai R. Edwards L. Kodavali C. Sherry Psy N. S. (2023). Human brain organoid code of conduct. *Front. Mol. Med.* 3:1143298. 10.3389/FMMED.2023.1143298 39086687 PMC11285598

[B111] Huang X. Huang Z. Gao W. Gao W. He R. Li Y. (2022). Current advances in 3D dynamic cell culture systems. *Gels* 8:829. 10.3390/GELS8120829 36547353 PMC9778081

[B112] Hughes J. P. Rees S. S. Kalindjian S. B. Philpott K. L. (2011). ‘Principles of early drug discovery’. *Br. J. Pharmacol.* 162 1239–1249. 10.1111/J.1476-5381.2010.01127.X 21091654 PMC3058157

[B113] Hyun I. Scharf-Deering J. C. Sullivan S. Aach J. D. Arlotta P. Baum M. L. (2022). ‘How collaboration between bioethicists and neuroscientists can advance research’. *Nat. Neurosci.* 25 1399–1401. 10.1038/S41593-022-01187-2 36258039

[B114] Infante G. Miceli R. Ambrogi F. (2023). ‘Sample size and predictive performance of machine learning methods with survival data: a simulation study’. *Stat. Med.* 42 5657–5675. 10.1002/SIM.9931 37947168

[B115] Ishii M. N. Yamamoto K. Shoji M. Asami A. Kawamata Y. (2017). ‘Human induced pluripotent stem cell (hiPSC)-derived neurons respond to convulsant drugs when co-cultured with hiPSC-derived astrocytes’. *Toxicology* 389 130–138. 10.1016/J.TOX.2017.06.010 28666936

[B116] Jang M. J. Nam Y. (2015). ‘NeuroCa: integrated framework for systematic analysis of spatiotemporal neuronal activity patterns from large-scale optical recording data’. *Neurophotonics* 2:035003. 10.1117/1.NPH.2.3.035003 26229973 PMC4516777

[B117] Jensen C. Teng Y. (2020). Is it time to start transitioning from 2D to 3D cell culture? *Front. Mol. Biosci.* 7:33. 10.3389/FMOLB.2020.00033 32211418 PMC7067892

[B118] Jin L. Han Z. Platisa J. Wooltorton J. R. A. Cohen L. B. Pieribone V. A. (2012). ‘Single action potentials and subthreshold electrical events imaged in neurons with a fluorescent protein voltage probe’. *Neuron* 75 779–785. 10.1016/J.NEURON.2012.06.040 22958819 PMC3439164

[B119] Jo J. Xiao Y. Sun A. X. Cukuroglu E. Tran H. D. Göke J. (2016). ‘Midbrain-like organoids from human pluripotent stem cells contain functional dopaminergic and neuromelanin-producing neurons’. *Cell Stem Cell* 19 248–257. 10.1016/J.STEM.2016.07.005 27476966 PMC5510242

[B120] Jones R. S. G. da Silva A. B. Whittaker R. G. Woodhall G. L. Cunningham M. O. (2016). ‘Human brain slices for epilepsy research: pitfalls, solutions and future challenges’. *J. Neurosci. Methods* 260 221–232. 10.1016/J.JNEUMETH.2015.09.021 26434706

[B121] Jourdon A. Wu F. Mariani J. Capauto D. Norton S. Tomasini L. (2023). ‘Modeling idiopathic autism in forebrain organoids reveals an imbalance of excitatory cortical neuron subtypes during early neurogenesis’. *Nat. Neurosci.* 26 1505–1515. 10.1038/S41593-023-01399-0 37563294 PMC10573709

[B122] Jumper J. Evans R. Pritzel A. Green T. Figurnov M. Ronneberger O. (2021). ‘Highly accurate protein structure prediction with AlphaFold’. *Nature* 596 583–589. 10.1038/S41586-021-03819-2 34265844 PMC8371605

[B123] Kanemaru K. Suzuki J. Taiko I. Iino M. (2020). Red fluorescent CEPIA indicators for visualization of Ca2+ dynamics in mitochondria. *Sci. Rep.* 10:2835. 10.1038/S41598-020-59707-8 32071363 PMC7029041

[B124] Kang Y. Zhou Y. Li Y. Han Y. Xu J. Niu W. (2021). ‘A human forebrain organoid model of fragile X syndrome exhibits altered neurogenesis and highlights new treatment strategies’. *Nat. Neurosci.* 24 1377–1391. 10.1038/S41593-021-00913-6 34413513 PMC8484073

[B125] Kannan M. Vasan G. Pieribone V. A. (2019). ‘Optimizing Strategies for Developing Genetically Encoded Voltage Indicators’. *Front. Cell. Neurosci.* 13:53. 10.3389/FNCEL.2019.00053 30863283 PMC6399427

[B126] Keller R. Basta R. Salerno L. Elia M. (2017). ‘Autism, epilepsy, and synaptopathies: a not rare association’. *Neurol. Sci.* 38 1353–1361. 10.1007/S10072-017-2974-X 28455770

[B127] Kiani A. K. Pheby D. Henehan G. Brown R. Sieving P. Sykora P. (2022). ‘Ethical considerations regarding animal experimentation’. *J. Prev. Med. Hyg.* 63 E255–E266. 10.15167/2421-4248/JPMH2022.63.2S3.2768 36479489 PMC9710398

[B128] Kiepas A. Voorand E. Mubaid F. Siegel P. M. Brown C. M. (2020). Optimizing live-cell fluorescence imaging conditions to minimize phototoxicity. *J. Cell Sci.* 133:jcs242834. 10.1242/JCS.242834 31988150

[B129] Kilfoil P. Feng S. L. Bassyouni A. Lee T. Leishman D. Li D. (2021). ‘Characterization of a high throughput human stem cell cardiomyocyte assay to predict drug-induced changes in clinical electrocardiogram parameters’. *Eur. J. Pharmacol.* 912:174584. 10.1016/J.EJPHAR.2021.174584 34678241

[B130] Kim J. Imaizumi K. Thete M. V. Hudacova Z. Jurjuţ O. Amin N. D. (2024). Human assembloid model of the ascending neural sensory pathway. *bioRxiv [Preprint]* 10.1101/2024.03.11.584539 38559133 PMC10979925

[B131] Kiskinis E. Kralj J. M. Zou P. Weinstein E. N. Zhang H. Tsioras K. (2018). ‘All-optical electrophysiology for high-throughput functional characterization of a human iPSC-derived motor neuron model of ALS’. *Stem Cell Rep.* 10 1991–2004. 10.1016/J.STEMCR.2018.04.020 29779896 PMC5993648

[B132] Klöppel S. Stonnington C. M. Chu C. Draganski B. Scahill R. I. Rohrer J. D. (2008). ‘Automatic classification of MR scans in Alzheimer’s disease’. *Brain* 131(Pt. 3), 681–689. 10.1093/BRAIN/AWM319 18202106 PMC2579744

[B133] Knöpfel T. Song C. (2019). ‘Optical voltage imaging in neurons: moving from technology development to practical tool’. *Nat. Rev. Neurosci.* 20 719–727. 10.1038/S41583-019-0231-4 31705060

[B134] Kolar K. Dondorp D. Zwiggelaar J. C. Høyer J. Chatzigeorgiou M. (2021). Mesmerize is a dynamically adaptable user-friendly analysis platform for 2D and 3D calcium imaging data. *Nat. Commun.* 12:6569. 10.1038/S41467-021-26550-Y 34772921 PMC8589933

[B135] Kolb I. Landry C. R. Yip M. C. Lewallen C. F. Stoy W. A. Lee J. (2019). PatcherBot: a single-cell electrophysiology robot for adherent cells and brain slices. *J. Neural Eng.* 16:046003. 10.1088/1741-2552/AB1834 30970335 PMC7144284

[B136] Koos K. Oláh G. Balassa T. Mihut N. Rózsa M. Ozsvár A. (2021). Automatic deep learning-driven label-free image-guided patch clamp system. *Nat. Commun.* 12:936. 10.1038/S41467-021-21291-4 33568670 PMC7875980

[B137] Kuijlaars J. Oyelami T. Diels A. Rohrbacher J. Versweyveld S. Meneghello G. (2016). Sustained synchronized neuronal network activity in a human astrocyte co-culture system. *Sci. Rep.* 6:36529. 10.1038/SREP36529 27819315 PMC5098163

[B138] Kularatna S. Jadambaa A. Senanayake S. Brain D. Hawker N. Kasparian N. A. (2022). ‘The cost of neurodevelopmental disability: scoping review of economic evaluation methods’. *Clin. Econ. Outcomes Res.* 14 665–682. 10.2147/CEOR.S370311 36304697 PMC9596191

[B139] Kulkarni R. U. Miller E. W. (2017). ‘Voltage imaging: pitfalls and potential’. *Biochemistry* 56 5171–5177. 10.1021/ACS.BIOCHEM.7B00490 28745864 PMC5715730

[B140] Lancaster M. A. Knoblich J. A. (2014). ‘Generation of cerebral organoids from human pluripotent stem cells’. *Nat. Protoc.* 9 2329–2340. 10.1038/NPROT.2014.158 25188634 PMC4160653

[B141] Lancaster M. A. Renner M. Martin C. A. Wenzel D. Bicknell L. S. Hurles M. E. (2013). ‘Cerebral organoids model human brain development and microcephaly’. *Nature* 501 373–379. 10.1038/NATURE12517 23995685 PMC3817409

[B142] Landry C. R. Yip M. C. Zhou Y. Niu W. Wang Y. Yang B. (2023). Electrophysiological and morphological characterization of single neurons in intact human brain organoids. *J. Neurosci. Methods* 394:109898. 10.1016/J.JNEUMETH.2023.109898 37236404 PMC10483933

[B143] Lascano A. M. Korff C. M. Picard F. (2016). ‘Seizures and epilepsies due to channelopathies and neurotransmitter receptor dysfunction: a parallel between genetic and immune aspects’. *Mol. Syndromol.* 7 197–209. 10.1159/000447707 27781030 PMC5073503

[B144] Lee E. Lee J. Kim E. (2017). ‘Excitation/inhibition imbalance in animal models of autism spectrum disorders’. *Biol. Psychiatry* 81 838–847. 10.1016/J.BIOPSYCH.2016.05.011 27450033

[B145] Lee J. Ha S. Ahn J. Lee S. T. Choi J. R. Cheon K. A. (2021). The role of ion channel-related genes in autism spectrum disorder: a study using next-generation sequencing. *Front. Genet.* 12:595934. 10.3389/FGENE.2021.595934 34712263 PMC8546317

[B146] Levakov G. Rosenthal G. Shelef I. Raviv T. R. Avidan G. (2020). ‘From a deep learning model back to the brain-Identifying regional predictors and their relation to aging’. *Human Brain Mapp.* 41 3235–3252. 10.1002/HBM.25011 32320123 PMC7426775

[B147] Leyrer-Jackson J. M. Olive M. F. Gipson C. D. (2019). ‘Whole-cell patch-clamp electrophysiology to study ionotropic glutamatergic receptors and their roles in addiction’. *Methods Mol. Biol. (Clifton, N.J.)* 1941 107–135. 10.1007/978-1-4939-9077-1_9 30707431 PMC6642821

[B148] Lin H. C. He Z. Ebert S. Schörnig M. Santel M. Nikolova M. T. (2021). ‘NGN2 induces diverse neuron types from human pluripotency’. *Stem Cell Rep.* 16 2118–2127. 10.1016/J.STEMCR.2021.07.006 34358451 PMC8452516

[B149] Lippert M. T. Takagaki K. Xu W. Huang X. Wu J. Y. (2007). ‘Methods for voltage-sensitive dye imaging of rat cortical activity with high signal-to-noise ratio’. *J. Neurophysiol.* 98 502–512. 10.1152/JN.01169.2006 17493915 PMC2855339

[B150] Liu C. Oikonomopoulos A. Sayed N. Wu J. C. (2018). Modeling human diseases with induced pluripotent stem cells: from 2D to 3D and beyond. *Development (Cambridge)* 145:dev156166. 10.1242/DEV.156166 29519889 PMC5868991

[B151] Liu Y. Chen X. Ma Y. Song C. Ma J. Chen C. (2024). Endogenous mutant Huntingtin alters the corticogenesis via lowering Golgi recruiting ARF1 in cortical organoid. *Mol. Psychiatry* 29 3024–3039. 10.1038/S41380-024-02562-0 38654124 PMC11449793

[B152] Lu H. R. Seo M. Kreir M. Tanaka T. Yamoto R. Altrocchi C. (2023). High-throughput screening assay for detecting drug-induced changes in synchronized neuronal oscillations and potential seizure risk based on Ca2+ fluorescence measurements in human induced pluripotent stem cell (hiPSC)-derived neuronal 2D And 3D cultures. *Cells* 12:958. 10.3390/CELLS12060958 36980298 PMC10046961

[B153] Ludwig T. E. Andrews P. W. Barbaric I. Benvenisty N. Bhattacharyya A. Crook J. M. (2023). ‘ISSCR standards for the use of human stem cells in basic research’. *Stem Cell Rep.* 18 1744–1752. 10.1016/j.stemcr.2023.08.003 37703820 PMC10545481

[B154] Marchetto M. C. N. Carromeu C. Acab A. Yu D. Yeo G. W. Mu Y. (2010). ‘A model for neural development and treatment of Rett syndrome using human induced pluripotent stem cells’. *Cell* 143 527–539. 10.1016/J.CELL.2010.10.016 21074045 PMC3003590

[B155] Mariani J. Simonini M. V. Palejev D. Tomasini L. Coppola G. Szekely A. M. (2012). ‘Modeling human cortical development in vitro using induced pluripotent stem cells’. *Proc. Natl Acad. Sci. U.S.A.* 109 12770–12775. 10.1073/PNAS.1202944109 22761314 PMC3411972

[B156] Martinez-Curiel R. Jansson L. Tsupykov O. Avaliani N. Aretio-Medina C. Hidalgo I. (2023). ‘Oligodendrocytes in human induced pluripotent stem cell-derived cortical grafts remyelinate adult rat and human cortical neurons’. *Stem Cell Rep.* 18 1643–1656. 10.1016/J.STEMCR.2023.04.010 37236198 PMC10444570

[B157] Matsuda N. Odawara A. Kinoshita K. Okamura A. Shirakawa T. Suzuki I. (2022). Raster plots machine learning to predict the seizure liability of drugs and to identify drugs. *Sci. Rep.* 12:2281. 10.1038/S41598-022-05697-8 35145132 PMC8831568

[B158] McCready F. P. Gordillo-Sampedro S. Pradeepan K. Martinez-Trujillo J. Ellis J. (2022). Multielectrode arrays for functional phenotyping of neurons from induced pluripotent stem cell models of neurodevelopmental disorders. *Biology* 11:316. 10.3390/BIOLOGY11020316 35205182 PMC8868577

[B159] Mederos S. González-Arias C. Perea G. (2018). Astrocyte–neuron networks: a multilane highway of signaling for homeostatic brain function. *Front. Synaptic Neurosci.* 10:45. 10.3389/FNSYN.2018.00045 30542276 PMC6277918

[B160] Milosevic M. M. Jang J. McKimm E. J. Zhu M. H. Antic S. D. (2020). ‘In vitro testing of voltage indicators: Archon1, arclightd, asap1, asap2s, asap3b, bongwoori-pos6, berst1, flicr1, and chi-vsfp-butterfly. *eNeuro* 7 ENEURO.0060-20.2020. 10.1523/ENEURO.0060-20.2020 32817120 PMC7540930

[B161] Miura Y. Li M. Y. Birey F. Ikeda K. Revah O. Thete M. V. (2020). Generation of human striatal organoids and cortico-striatal assembloids from human pluripotent stem cells. *Nat. Biotechnol.* 38:1421. 10.1038/S41587-020-00763-W 33273741 PMC9042317

[B162] Mohajeri K. Yadav R. D’haene E. Boone P. M. Erdin S. Gao D. (2022). ‘Transcriptional and functional consequences of alterations to MEF2C and its topological organization in neuronal models’. *Am. J. Hum. Genet.* 109 2049–2067. 10.1016/J.AJHG.2022.09.015 36283406 PMC9674968

[B163] Mok R. S. F. Zhang W. Sheikh T. I. Pradeepan K. Fernandes I. R. DeJong L. C. (2022). ‘Wide spectrum of neuronal and network phenotypes in human stem cell-derived excitatory neurons with Rett syndrome-associated MECP2 mutations’. *Transl. Psychiatry.* 12, 1–16. 10.1038/s41398-022-02216-1 36253345 PMC9576700

[B164] Moreau C. Deruelle C. Auzias G. (2023). ‘Machine learning for neurodevelopmental disorders’. *Neuromethods* 197 977–1007. 10.1007/978-1-0716-3195-9_31 37988540

[B165] Mossink B. Verboven A. H. A. van Hugte E. J. H. Klein Gunnewiek T. M. Parodi G. Linda K. (2021). ‘Human neuronal networks on micro-electrode arrays are a highly robust tool to study disease-specific genotype-phenotype correlations in vitro’. *Stem Cell Rep.* 16 2182–2196. 10.1016/J.STEMCR.2021.07.001 34329594 PMC8452490

[B166] Movaghar A. Page D. Brilliant M. Mailick M. (2022). Advancing artificial intelligence-assisted pre-screening for fragile X syndrome. *BMC Med. Inform. Decis. Making* 22:152. 10.1186/S12911-022-01896-5 35689224 PMC9185893

[B167] Mullin A. P. Gokhale A. Moreno-De-Luca A. Sanyal S. Waddington J. L. Faundez V. (2013). Neurodevelopmental disorders: mechanisms and boundary definitions from genomes, interactomes and proteomes. *Transl. Psychiatry* 3:e329. 10.1038/TP.2013.108 24301647 PMC4030327

[B168] Muzzi L. Di Lisa D. Falappa M. Pepe S. Maccione A. Pastorino L. (2023). Human-derived cortical neurospheroids coupled to passive, high-density and 3D MEAs: a valid platform for functional tests. *Bioengineering (Basel, Switzerland)* 10:449. 10.3390/BIOENGINEERING10040449 37106636 PMC10136157

[B169] Nageshappa S. Carromeu C. Trujillo C. A. Mesci P. Espuny-Camacho I. Pasciuto E. (2016). ‘Altered neuronal network and rescue in a human MECP2 duplication model’. *Mol. Psychiatry* 21 178–188. 10.1038/MP.2015.128 26347316 PMC4720528

[B170] Negraes P. D. Trujillo C. A. Yu N. K. Wu W. Yao H. Liang N. (2021). ‘Altered network and rescue of human neurons derived from individuals with early-onset genetic epilepsy’. *Mol. Psychiatry* 26 7047–7068. 10.1038/S41380-021-01104-2 33888873 PMC8531162

[B171] Neher E. Sakmann B. (1976). ‘Single-channel currents recorded from membrane of denervated frog muscle fibres’. *Nature* 260 799–802. 10.1038/260799A0 1083489

[B172] Nehme R. Zuccaro E. Ghosh S. D. Li C. Sherwood J. L. Pietilainen O. (2018). ‘Combining NGN2 programming with developmental patterning generates human excitatory neurons with NMDAR-mediated synaptic transmission’. *Cell Rep.* 23 2509–2523. 10.1016/J.CELREP.2018.04.066 29791859 PMC6003669

[B173] Nguyen C. Upadhyay H. Murphy M. Borja G. Rozsahegyi E. J. Barnett A. (2019). ‘Simultaneous voltage and calcium imaging and optogenetic stimulation with high sensitivity and a wide field of view’. *Biomed. Opt. Express* 10:789. 10.1364/BOE.10.000789 30800515 PMC6377900

[B174] Norori N. Hu Q. Aellen F. M. Faraci F. D. Tzovara A. (2021). Addressing bias in big data and AI for health care: a call for open science. *Patterns (New York, N.Y.)* 2:100347. 10.1016/J.PATTER.2021.100347 34693373 PMC8515002

[B175] Obergrussberger A. Brüggemann A. Goetze T. A. Rapedius M. Haarmann C. Rinke I. (2016). ‘Automated patch clamp meets high-throughput screening: 384 cells recorded in parallel on a planar patch clamp module’. *J. Lab. Autom.* 21 779–793. 10.1177/2211068215623209 26702021

[B176] Obien M. E. J. Deligkaris K. Bullmann T. Bakkum D. J. Frey U. (2015). ‘Revealing neuronal function through microelectrode array recordings’. *Front. Neurosci.* 8:423. 10.3389/FNINS.2014.00423 25610364 PMC4285113

[B177] Pachitariu M. Stringer C. (2022). ‘Cellpose 2.0: how to train your own model’. *Nat. Methods* 19 1634–1641. 10.1038/S41592-022-01663-4 36344832 PMC9718665

[B178] Pachitariu M. Steinmetz N. Kadir S. Carandini M. Harris K. D. (2016). Kilosort: realtime spike-sorting for extracellular electrophysiology with hundreds of channels. *bioRxiv [Preprint]* 10.1101/061481

[B179] Pankevich D. E. Altevogt B. M. Dunlop J. Gage F. H. Hyman S. E. (2014). ‘Improving and accelerating drug development for nervous system disorders’. *Neuron* 84 546–553. 10.1016/J.NEURON.2014.10.007 25442933 PMC4254615

[B180] Parenti I. Rabaneda L. G. Schoen H. Novarino G. (2020). ‘Neurodevelopmental disorders: from genetics to functional pathways’. *Trends Neurosci.* 43 608–621. 10.1016/J.TINS.2020.05.004 32507511

[B181] Parnell E. Culotta L. Forrest M. P. Jalloul H. A. Eckman B. L. Loizzo D. D. (2023). ‘Excitatory dysfunction drives network and calcium handling deficits in 16p11.2 duplication schizophrenia induced pluripotent stem cell-derived neurons’. *Biol. Psychiatry* 94 153–163. 10.1016/J.BIOPSYCH.2022.11.005 36581494 PMC10166768

[B182] Parodi G. Brofiga M. Pastore V. P. Chiappalone M. Martinoia S. (2023). Deepening the role of excitation/inhibition balance in human iPSCs-derived neuronal networks coupled to MEAs during long-term development. *J. Neural Eng.* 20 1–18. 10.1088/1741-2552/ACF78B 37678214

[B183] Pasca A. M. Sloan S. A. Clarke L. E. Tian Y. Makinson C. D. Huber N. (2015). ‘Functional cortical neurons and astrocytes from human pluripotent stem cells in 3D culture’. *Nat. Methods* 12 671–678. 10.1038/NMETH.3415 26005811 PMC4489980

[B184] Pas̨ca S. P. (2024). Constructing human neural circuits in living systems by transplantation. *Cell* 187 8–13. 10.1016/J.CELL.2023.12.008 38181744

[B185] Pavinato L. Delle Vedove A. Carli D. Ferrero M. Carestiato S. Howe J. L. (2023). ‘CAPRIN1 haploinsufficiency causes a neurodevelopmental disorder with language impairment, ADHD and ASD. *Brain’* 146 534–548. 10.1093/BRAIN/AWAC278 35979925 PMC10169411

[B186] Perea G. Sur M. Araque A. (2014). ‘Neuron-glia networks: integral gear of brain function’. *Front. Cell. Neurosci.* 8:378. 10.3389/FNCEL.2014.00378 25414643 PMC4222327

[B187] Perich M. G. Rajan K. (2020). ‘Rethinking brain-wide interactions through multi-region “network of networks” models’. *Curr. Opin. Neurobiol.* 65 146–151. 10.1016/J.CONB.2020.11.003 33254073 PMC7822595

[B188] Perich M. G. Arlt C. Soares S. Young M. E. Mosher C. P. Minxha J. (2021). Inferring brain-wide interactions using data-constrained recurrent neural network models. *bioRxiv [Preprint]* 10.1101/2020.12.18.423348 PMC13565020 42567158

[B189] Perszyk R. E. Yip M. C. McConnell O. L. Wang E. T. Jenkins A. Traynelis S. F. (2021). ‘Automated intracellular pharmacological electrophysiology for ligand-gated ionotropic receptor and pharmacology screening’. *Mol. Pharmacol.* 100 73–82. 10.1124/MOLPHARM.120.000195 33958481 PMC8274318

[B190] Peterka D. S. Takahashi H. Yuste R. (2011). ‘Imaging voltage in neurons’. *Neuron* 69 9–21. 10.1016/J.NEURON.2010.12.010 21220095 PMC3387979

[B191] Pologruto T. A. Yasuda R. Svoboda K. (2004). ‘Monitoring neural activity and [Ca2+] with genetically encoded Ca2+ indicators’. *J. Neurosci.’* 24 9572–9579. 10.1523/JNEUROSCI.2854-04.2004 15509744 PMC6730159

[B192] Porciúncula L. O. Goto-Silva L. Ledur P. F. Rehen S. K. (2021). The age of brain organoids: tailoring cell identity and functionality for normal brain development and disease modeling. *Front. Neurosci.* 15:674563. 10.3389/FNINS.2021.674563 34483818 PMC8414411

[B193] Pradeepan K. S. McCready F. P. Wei W. Khaki M. Zhang W. Salter M. W. (2024). Calcium-dependent hyperexcitability in human stem cell-derived rett syndrome neuronal networks. *Biol. Psychiatry Glob. Open Sci.* 4:100290. 10.1016/J.BPSGOS.2024.100290 38420187 PMC10899066

[B194] Puppo F. Sadegh S. Trujillo C. A. Thunemann M. Campbell E. P. Vandenberghe M. (2021). All-optical electrophysiology in hiPSC-derived neurons with synthetic voltage sensors. *Front. Cell. Neurosci.* 15:671549. 10.3389/FNCEL.2021.671549 34122014 PMC8193062

[B195] Qi Y. Zhang X. J. Renier N. Wu Z. Atkin T. Sun Z. (2017). ‘Combined small-molecule inhibition accelerates the derivation of functional cortical neurons from human pluripotent stem cells’. *Nat. Biotechnol.* 35 154–163. 10.1038/NBT.3777 28112759 PMC5516899

[B196] Qu G. Merchant J. P. Clatot J. DeFlitch L. M. Frederick D. J. Tang S. (2024). ‘Targeted blockade of aberrant sodium current in a stem cell-derived neuron model of SCN3A encephalopathy’. *Brain* 147 1247–1263. 10.1093/BRAIN/AWAD376 37935051 PMC10994535

[B197] Quadrato G. Nguyen T. Macosko E. Z. Sherwood J. L. Yang S. M. Berger D. R. (2017). ‘Cell diversity and network dynamics in photosensitive human brain organoids’. *Nature* 545 48–53. 10.1038/NATURE22047 28445462 PMC5659341

[B198] Quraishi I. H. Stern S. Mangan K. P. Zhang Y. Ali S. R. Mercier M. R. (2019). ‘An epilepsy-associated KCNT1 mutation enhances excitability of human iPSC-derived neurons by increasing slack KNa currents’. *J. Neurosci.’* 39 7438–7449. 10.1523/JNEUROSCI.1628-18.2019 31350261 PMC6759030

[B199] Rajput D. Wang W. J. Chen C. C. (2023). Evaluation of a decided sample size in machine learning applications. *BMC Bioinform.* 24:48. 10.1186/S12859-023-05156-9 36788550 PMC9926644

[B200] Reel P. S. Reel S. Pearson E. Trucco E. Jefferson E. (2021). Using machine learning approaches for multi-omics data analysis: a review. *Biotechnol. Adv.* 49:107739. 10.1016/J.BIOTECHADV.2021.107739 33794304

[B201] Revah O. Gore F. Kelley K. W. Andersen J. Sakai N. Chen X. (2022). ‘Maturation and circuit integration of transplanted human cortical organoids’. *Nature* 610 319–326. 10.1038/S41586-022-05277-W 36224417 PMC9556304

[B202] Richter-Laskowska M. Trybek P. Bednarczyk P. Wawrzkiewicz-Jałowiecka A. (2021). ‘Application of machine-learning methods to recognize mitobk channels from different cell types based on the experimental patch-clamp results’. *Int. J. Mol. Sci.* 22:840. 10.3390/IJMS22020840 33467711 PMC7831025

[B203] Rossi G. Giger S. Hübscher T. Lutolf M. P. (2022). Gastruloids as in vitro models of embryonic blood development with spatial and temporal resolution. *Sci. Rep.* 12:13380. 10.1038/S41598-022-17265-1 35927563 PMC9352713

[B204] Russo F. B. Freitas B. C. Pignatari G. C. Fernandes I. R. Sebat J. Muotri A. R. (2018). ‘Modeling the interplay between neurons and astrocytes in autism using human induced pluripotent stem cells’. *Biol. Psychiatry* 83 569–578. 10.1016/J.BIOPSYCH.2017.09.021 29129319

[B205] Rylaarsdam L. Rakotomamonjy J. Pope E. Guemez-Gamboa A. (2024). iPSC-derived models of PACS1 syndrome reveal transcriptional and functional deficits in neuron activity. *Nat. Commun.* 15:827. 10.1038/S41467-024-44989-7 38280846 PMC10821916

[B206] Saber W. A. Gasparoli F. M. Dirks M. G. Gunn-Moore F. J. Antkowiak M. (2018). All-optical assay to study biological neural networks. *Front. Neurosci.* 12:451. 10.3389/FNINS.2018.00451 30026684 PMC6041400

[B207] Sakaguchi H. Ozaki Y. Ashida T. Matsubara T. Oishi N. Kihara S. (2019). ‘Self-organized synchronous calcium transients in a cultured human neural network derived from cerebral organoids’. *Stem Cell Rep.* 13 458–473. 10.1016/J.STEMCR.2019.05.029 31257131 PMC6739638

[B208] Samarasinghe R. A. Miranda O. A. Buth J. E. Mitchell S. Ferando I. Watanabe M. (2021). ‘Identification of neural oscillations and epileptiform changes in human brain organoids’. *Nat. Neurosci.* 24 1488–1500. 10.1038/S41593-021-00906-5 34426698 PMC9070733

[B209] Sandoval S. O. Cappuccio G. Kruth K. Osenberg S. Khalil S. M. Méndez-Albelo N. M. (2024). ‘Rigor and reproducibility in human brain organoid research: where we are and where we need to go’. *Stem Cell Rep.* 19 796–816. 10.1016/J.STEMCR.2024.04.008 38759644 PMC11297560

[B210] Schwarz N. Uysal B. Welzer M. Bahr J. C. Layer N. Löffler H. (2019). Long-term adult human brain slice cultures as a model system to study human CNS circuitry and disease. *eLife* 8:e48417. 10.7554/ELIFE.48417 31498083 PMC6733599

[B211] Sebastian J. Sur M. Murthy H. A. Magimai-Doss M. (2021). Signal-to-signal neural networks for improved spike estimation from calcium imaging data. *PLoS Comput. Biol.* 17:e1007921. 10.1371/JOURNAL.PCBI.1007921 33647015 PMC7951974

[B212] Seeman S. C. Campagnola L. Davoudian P. A. Hoggarth A. Hage T. A. Bosma-Moody A. (2018). Sparse recurrent excitatory connectivity in the microcircuit of the adult mouse and human cortex. *eLife* 7:e37349. 10.7554/ELIFE.37349 30256194 PMC6158007

[B213] Segev A. Garcia-Oscos F. Kourrich S. (2016). Whole-cell patch-clamp recordings in brain slices. *J. Visual. Exp.* 112:54024. 10.3791/54024 27341060 PMC4927800

[B214] Seibertz F. Rapedius M. Fakuade F. E. Tomsits P. Liutkute A. Cyganek L. (2022). A modern automated patch-clamp approach for high throughput electrophysiology recordings in native cardiomyocytes. *Commun. Biol.* 5:969. 10.1038/S42003-022-03871-2 36109584 PMC9477872

[B215] Shan X. Zhang A. Rezzonico M. G. Tsai M.-C. Sanchez-Priego C. Zhang Y. (2024). ‘Fully defined NGN2 neuron protocol reveals diverse signatures of neuronal maturation’. *Cell Rep. Methods* 4:100858. 10.1016/j.crmeth.2024.100858 39255791 PMC11440061

[B216] Shapson-Coe A. Januszewski M. Berger D. R. Pope A. Wu Y. Blakely T. (2024). ‘A petavoxel fragment of human cerebral cortex reconstructed at nanoscale resolution’. *Science (New York, N.Y.)* 384:eadk4858. 10.1126/SCIENCE.ADK4858 38723085 PMC11718559

[B217] Sharf T. van der Molen T. Glasauer S. M. K. Guzman E. Buccino A. P. Luna G. (2022). Functional neuronal circuitry and oscillatory dynamics in human brain organoids. *Nat. Commun.* 13:4403. 10.1038/S41467-022-32115-4 35906223 PMC9338020

[B218] Sharma S. D. Reddy B. K. Pal R. Ritakari T. E. Cooper J. D. Selvaraj B. T. (2023). Astrocytes mediate cell non-autonomous correction of aberrant firing in human FXS neurons. *Cell Rep.* 42:112344. 10.1016/J.CELREP.2023.112344 37018073 PMC10157295

[B219] Shen W. Pristov J. Nobili P. Nikoliæ L. (2023). ‘Can glial cells save neurons in epilepsy?’. *Neural Regener. Res.* 18 1417–1422. 10.4103/1673-5374.360281 36571336 PMC10075109

[B220] Simkin D. Marshall K. A. Vanoye C. G. Desai R. R. Bustos B. I. Piyevsky B. N. (2021). ‘Dyshomeostatic modulation of Ca2+-activated K+ channels in a human neuronal model of KCNQ2 encephalopathy. *eLife* 10:e64434. 10.7554/ELIFE.64434 33544076 PMC7864629

[B221] Sirenko O. Parham F. Dea S. Sodhi N. Biesmans S. Mora-Castilla S. (2019). ‘Functional and mechanistic neurotoxicity profiling using human iPSC-derived neural 3d cultures’. *Toxicol. Sci.* 167 249–257. 10.1093/TOXSCI/KFY218 30169818 PMC6317428

[B222] Smith A. S. T. Choi E. Gray K. Macadangdang J. Ahn E. H. Clark E. C. (2020). ‘NanoMEA: a tool for high-throughput, electrophysiological phenotyping of patterned excitable cells. *Nano Lett.* 20 1561–1570. 10.1021/ACS.NANOLETT.9B04152 31845810 PMC7547911

[B223] Song C. Jiang Z. Q. Liu D. Wu L. L. (2022). Application and research progress of machine learning in the diagnosis and treatment of neurodevelopmental disorders in children. *Front. Psychiatry* 13:960672. 10.3389/FPSYT.2022.960672 36090350 PMC9449316

[B224] Sperandeo A. Tamburini C. Noakes Z. De La Fuente D. C. Keefe F. Petter O. (2023). ‘Cortical neuronal hyperexcitability and synaptic changes in SGCE mutation-positive myoclonus dystonia’. *Brain* 146 1523–1541. 10.1093/BRAIN/AWAC365 36204995 PMC10115238

[B225] Stafstrom C. E. Carmant L. (2015). ‘Seizures and epilepsy: an overview for neuroscientists’. *Cold Spring Harb. Perspect. Biol.* 5:a022426. 10.1101/CSHPERSPECT.A022426 26033084 PMC4448698

[B226] Stringer C. Pachitariu M. (2019). ‘Computational processing of neural recordings from calcium imaging data’. *Curr. Opin. Neurobiol.* 55 22–31. 10.1016/J.CONB.2018.11.005 30530255

[B227] Sukoff Rizzo S. J. Crawley J. N. (2017). ‘Behavioral phenotyping assays for genetic mouse models of neurodevelopmental, neurodegenerative, and psychiatric disorders. *Annu. Rev. Anim. Biosci.* 5 371–389. 10.1146/ANNUREV-ANIMAL-022516-022754 28199172

[B228] Sun A. X. Yuan Q. Fukuda M. Yu W. Yan H. Lim G. G. Y. (2019). ‘Potassium channel dysfunction in human neuronal models of Angelman syndrome’. *Science (New York, N.Y.)* 366 1486–1492. 10.1126/SCIENCE.AAV5386 31857479 PMC7735558

[B229] Sun D. Gao W. Hu H. Zhou S. (2022). ‘Why 90% of clinical drug development fails and how to improve it?’. *Acta Pharm. Sin. B* 12 3049–3062. 10.1016/J.APSB.2022.02.002 35865092 PMC9293739

[B230] Sun J. Osenberg S. Irwin A. Ma L. H. Lee N. Xiang Y. (2023). Mutations in the transcriptional regulator MeCP2 severely impact key cellular and molecular signatures of human astrocytes during maturation. *Cell Rep.* 42:111942. 10.1016/J.CELREP.2022.111942 36640327 PMC10857774

[B231] Sundberg M. Pinson H. Smith R. S. Winden K. D. Venugopal P. Tai D. J. C. (2021). 16p11.2 deletion is associated with hyperactivation of human iPSC-derived dopaminergic neuron networks and is rescued by RHOA inhibition in vitro. *Nat. Commun.* 12:2897. 10.1038/S41467-021-23113-Z 34006844 PMC8131375

[B232] Supakul S. Murakami R. Oyama C. Shindo T. Hatakeyama Y. Itsuno M. (2024). Mutual interaction of neurons and astrocytes derived from iPSCs with APP V717L mutation developed the astrocytic phenotypes of Alzheimer’s disease. *Inflamm. Regener.* 44:8. 10.1186/S41232-023-00310-5 38419091 PMC10900748

[B233] Susco S. G. Ghosh S. Mazzucato P. Angelini G. Beccard A. Barrera V. (2022). ‘Molecular convergence between Down syndrome and fragile X syndrome identified using human pluripotent stem cell models’. *Cell Rep.* 40:111312. 10.1016/J.CELREP.2022.111312 36070702 PMC9465809

[B234] Sussillo D. Churchland M. M. Kaufman M. T. Shenoy K. V. (2015). ‘A neural network that finds a naturalistic solution for the production of muscle activity’. *Nat. Neurosci.* 18 1025–1033. 10.1038/NN.4042 26075643 PMC5113297

[B235] Suzuki J. Kanemaru K. Iino M. (2016). ‘Genetically encoded fluorescent indicators for organellar calcium imaging’. *Biophys. J.* 111 1119–1131. 10.1016/J.BPJ.2016.04.054 27477268 PMC5034299

[B236] Suzuki J. Kanemaru K. Ishii K. Ohkura M. Okubo Y. Iino M. (2014). Imaging intraorganellar Ca2+ at subcellular resolution using CEPIA. *Nat. Commun.* 5:4153. 10.1038/NCOMMS5153 24923787 PMC4082642

[B237] Tada M. Takeuchi A. Hashizume M. Kitamura K. Kano M. (2014). ‘A highly sensitive fluorescent indicator dye for calcium imaging of neural activity in vitro and in vivo’. *Eur. J. Neurosci.* 39 1720–1728. 10.1111/EJN.12476 24405482 PMC4232931

[B238] Takahashi K. Tanabe K. Ohnuki M. Narita M. Ichisaka T. Tomoda K. (2007). ‘Induction of pluripotent stem cells from adult human fibroblasts by defined factors’. *Cell* 131 861–872. 10.1016/J.CELL.2007.11.019 18035408

[B239] Tang X. Kim J. Zhou L. Wengert E. Zhang L. Wu Z. (2016). ‘KCC2 rescues functional deficits in human neurons derived from patients with Rett syndrome’. *Proc. Natl Acad. Sci. U.S.A.* 113 751–756. 10.1073/PNAS.1524013113 26733678 PMC4725523

[B240] Tang X. Zhou L. Wagner A. M. Marchetto M. C. N. Muotri A. R. Gage F. H. (2013). ‘Astroglial cells regulate the developmental timeline of human neurons differentiated from induced pluripotent stem cells’. *Stem Cell Res.* 11 743–757. 10.1016/J.SCR.2013.05.002 23759711 PMC3979966

[B241] Telias M. Kuznitsov-Yanovsky L. Sega M. Ben-Yosef D. (2015). ‘Functional deficiencies in fragile X neurons derived from human embryonic stem cells’. *J. Neurosci.* 35 15295–15306. 10.1523/JNEUROSCI.0317-15.2015 26586818 PMC6605488

[B242] Tian Y. Wu X. Luo S. Xiong D. Liu R. Hu L. (2024). ‘A multi-omic single-cell landscape of cellular diversification in the developing human cerebral cortex’. *Comput. Struct. Biotechnol. J.* 23 2173–2189. 10.1016/J.CSBJ.2024.05.019 38827229 PMC11141146

[B243] Tidball A. M. Lopez-Santiago L. F. Yuan Y. Glenn T. W. Margolis J. L. Clayton Walker J. (2020). ‘Variant-specific changes in persistent or resurgent sodium current in SCN8A-related epilepsy patient-derived neurons’. *Brain* 143 3025–3040. 10.1093/BRAIN/AWAA247 32968789 PMC7780473

[B244] Tosheva K. L. Yuan Y. Matos Pereira P. Culley S. N. Henriques R. (2020). Between life and death: strategies to reduce phototoxicity in super-resolution microscopy. *J. Phys. D Appl. Phys.* 53:163001. 10.1088/1361-6463/AB6B95 33994582 PMC8114953

[B245] Trujillo C. A. Adams J. W. Negraes P. D. Carromeu C. Tejwani L. Acab A. (2021). Pharmacological reversal of synaptic and network pathology in human MECP2-KO neurons and cortical organoids. *EMBO Mol. Med.* 13:e12523. 10.15252/EMMM.202012523 33501759 PMC7799367

[B246] Trujillo C. A. Gao R. Negraes P. D. Gu J. Buchanan J. Preissl S. (2019). Complex oscillatory waves emerging from cortical organoids model early human brain network development. *Cell Stem Cell* 25 558–569.e7. 10.1016/J.STEM.2019.08.002 31474560 PMC6778040

[B247] Tsien R. Y. (1980). ‘New calcium indicators and buffers with high selectivity against magnesium and protons: design, synthesis, and properties of prototype structures’. *Biochemistry* 19 2396–2404. 10.1021/BI00552A018 6770893

[B248] Tukker A. M. Wijnolts F. M. J. de Groot A. Westerink R. H. S. (2018). ‘Human iPSC-derived neuronal models for in vitro neurotoxicity assessment’. *Neurotoxicology* 67 215–225. 10.1016/J.NEURO.2018.06.007 29909083

[B249] Urresti J. Zhang P. Moran-Losada P. Yu N. K. Negraes P. D. Trujillo C. A. (2021). ‘Cortical organoids model early brain development disrupted by 16p11.2 copy number variants in autism’. *Mol. Psychiatry* 26 7560–7580. 10.1038/S41380-021-01243-6 34433918 PMC8873019

[B250] Urrestizala-Arenaza N. Cerchio S. Cavaliere F. Magliaro C. (2024). ‘Limitations of human brain organoids to study neurodegenerative diseases: a manual to survive’. *Front. Cell. Neurosci.* 18:1419526. 10.3389/FNCEL.2024.1419526/BIBTEX PMC11267621 39049825

[B251] Vahsen B. F. Gray E. Candalija A. Cramb K. M. L. Scaber J. Dafinca R. (2022). Human iPSC co-culture model to investigate the interaction between microglia and motor neurons. *Sci. Rep.* 12:12606. 10.1038/S41598-022-16896-8 35871163 PMC9308778

[B252] Vakilzadeh G. Maseko B. C. Bartely T. D. McLennan Y. A. Martínez-Cerdeńo V. (2024). ‘Increased number of excitatory synapsis and decreased number of inhibitory synapsis in the prefrontal cortex in autism’. *Cereb. Cortex (New York, N.Y.’: 1991)* 34 121–128. 10.1093/CERCOR/BHAD268 38696601 PMC11065106

[B253] van Berkel A. A. Lammertse H. C. A. Öttl M. Koopmans F. Misra-Isrie M. Meijer M. (2023). ‘Reduced MUNC18-1 levels, synaptic proteome changes, and altered network activity in STXBP1-related disorder patient neurons. *Biol. Psychiatry Glob. Open Sci.* 4 284–298. 10.1016/J.BPSGOS.2023.05.004 38298782 PMC10829628

[B254] Van Hugte E. J. H. Lewerissa E. I. Wu K. M. Scheefhals N. Parodi G. Van Voorst T. W. (2023). ‘SCN1A-deficient excitatory neuronal networks display mutation-specific phenotypes’. *Brain* 146 5153–5167. 10.1093/BRAIN/AWAD245 37467479 PMC10689919

[B255] Vanoye C. G. Desai R. R. Ji Z. Adusumilli S. Jairam N. Ghabra N. (2022). High-throughput evaluation of epilepsy-associated KCNQ2 variants reveals functional and pharmacological heterogeneity. *JCI Insight* 7:e156314. 10.1172/JCI.INSIGHT.156314 35104249 PMC8983144

[B256] Varadi M. Anyango S. Deshpande M. Nair S. Natassia C. Yordanova G. (2022). AlphaFold protein structure database: massively expanding the structural coverage of protein-sequence space with high-accuracy models. *Nucleic Acids Res.* 50 D439–D444. 10.1093/NAR/GKAB1061 34791371 PMC8728224

[B257] Velasco S. Kedaigle A. J. Simmons S. K. Nash A. Rocha M. Quadrato G. (2019). ‘Individual brain organoids reproducibly form cell diversity of the human cerebral cortex’. *Nature* 570 523–527. 10.1038/S41586-019-1289-X 31168097 PMC6906116

[B258] Vijayalingam S. Ezekiel U. R. Xu F. Subramanian T. Geerling E. Hoelscher B. (2020). Human iPSC-derived neuronal cells From CTBP1-mutated patients reveal altered expression of neurodevelopmental gene networks. *Front. Neurosci.* 14:562292. 10.3389/FNINS.2020.562292 33192249 PMC7653094

[B259] Vijayan A. Diwakar S. (2022). A cerebellum inspired spiking neural network as a multi-model for pattern classification and robotic trajectory prediction. *Front. Neurosci.* 16:909146. 10.3389/FNINS.2022.909146 36518530 PMC9742384

[B260] Võfély G. Berecz T. Szabó E. Szebényi K. Hathy E. Orbán T. I. (2018). Characterization of calcium signals in human induced pluripotent stem cell-derived dentate gyrus neuronal progenitors and mature neurons, stably expressing an advanced calcium indicator protein. *Mol. Cell. Neurosci.* 88 222–230. 10.1016/J.MCN.2018.02.003 29425968

[B261] Voulgaris D. Nikolakopoulou P. Herland A. (2022). ‘Generation of Human iPSC-Derived Astrocytes with a mature star-shaped phenotype for CNS modeling’. *Stem Cell Rev. Rep.* 18 2494–2512. 10.1007/S12015-022-10376-2 35488987 PMC9489586

[B262] Wainger B. J. Kiskinis E. Mellin C. Wiskow O. Han S. S. W. Sandoe J. (2014). Intrinsic membrane hyperexcitability of amyotrophic lateral sclerosis patient-derived motor neurons. *Cell Rep.* 7 1–11. 10.1016/j.celrep.2014.03.019 24703839 PMC4023477

[B263] Walker A. S. Raliski B. K. Karbasi K. Zhang P. Sanders K. Miller E. W. (2021). Optical spike detection and connectivity analysis with a far-red voltage-sensitive fluorophore reveals changes to network connectivity in development and disease. *Front. Neurosci.* 15:643859. 10.3389/FNINS.2021.643859 34054405 PMC8155641

[B264] Wang Y. Chiola S. Yang G. Russell C. Armstrong C. J. Wu Y. (2022). Modeling human telencephalic development and autism-associated SHANK3 deficiency using organoids generated from single neural rosettes. *Nat. Commun.* 13:5688. 10.1038/S41467-022-33364-Z 36202854 PMC9537523

[B265] Werley C. A. Chien M.-P. Cohen A. E. (2017). ‘Ultrawidefield microscope for high-speed fluorescence imaging and targeted optogenetic stimulation’. *Biomed. Opt. Express* 8:5794. 10.1364/BOE.8.005794 29296505 PMC5745120

[B266] Whitaker M. (2010). Genetically encoded probes for measurement of intracellular calcium. *Methods Cell Biol.* 99 153–182. 10.1016/B978-0-12-374841-6.00006-2 21035686 PMC3292878

[B267] Whye D. Wood D. Saber W. A. Norabuena E. M. Makhortova N. R. Sahin M. (2023). A robust pipeline for the multi-stage accelerated differentiation of functional 3D cortical organoids from human pluripotent stem cells. *Curr. Protoc.* 3:e641. 10.1002/CPZ1.641 36633423 PMC9839317

[B268] Williams L. A. Gerber D. J. Elder A. Tseng W. C. Baru V. Delaney-Busch N. (2022). ‘Developing antisense oligonucleotides for a TECPR2 mutation-induced, ultra-rare neurological disorder using patient-derived cellular models’. *Mol. Ther. Nucleic Acids* 29:189. 10.1016/J.OMTN.2022.06.015 35860385 PMC9287140

[B269] Williams L. A. Joshi V. Murphy M. Ferrante J. Werley C. A. Brookings T. (2019). ‘Scalable measurements of intrinsic excitability in human iPS cell-derived excitatory neurons using all-optical electrophysiology’. *Neurochem. Res.* 44 714–725. 10.1007/S11064-018-2694-5 30603979

[B270] Williams L. A. Ryan S. J. Joshi V. Lewarch C. Elder A. McManus O. (2024). Discovery of novel compounds and target mechanisms using a high throughput, multiparametric phenotypic screen in a human neuronal model of Tuberous Sclerosis. *bioRxiv [Preprint]* 10.1101/2024.02.22.581652

[B271] Williamson R. C. Doiron B. Smith M. A. Yu B. M. (2019). ‘Bridging large-scale neuronal recordings and large-scale network models using dimensionality reduction’. *Curr. Opin. Neurobiol.* 55 40–47. 10.1016/J.CONB.2018.12.009 30677702 PMC6548625

[B272] Winden K. D. Pham T. T. Teaney N. A. Ruiz J. Chen R. Chen C. (2023). Increased degradation of FMRP contributes to neuronal hyperexcitability in tuberous sclerosis complex. *Cell Rep.* 42:112838. 10.1016/J.CELREP.2023.112838 37494191 PMC10529098

[B273] Winden K. D. Sundberg M. Yang C. Wafa S. M. A. Dwyer S. Chen P. F. (2019). ‘Biallelic mutations in TSC2 lead to abnormalities associated with cortical tubers in human ipsc-derived neurons’. *J. Neurosci.* 39 9294–9305. 10.1523/JNEUROSCI.0642-19.2019 31591157 PMC6867816

[B274] Woodruff G. Phillips N. Carromeu C. Guicherit O. White A. Johnson M. (2020). Screening for modulators of neural network activity in 3D human iPSC-derived cortical spheroids. *PLoS One* 15:e0240991. 10.1371/JOURNAL.PONE.0240991 33091047 PMC7581002

[B275] Wu W. Yao H. Negraes P. D. Wang J. Trujillo C. A. de Souza J. S. (2022). Neuronal hyperexcitability and ion channel dysfunction in CDKL5-deficiency patient iPSC-derived cortical organoids. *Neurobiol. Dis.* 174:105882. 10.1016/J.NBD.2022.105882 36202289 PMC13105298

[B276] Xue W. Li H. Xu J. Yu X. Liu L. Liu H. (2024). Effective cryopreservation of human brain tissue and neural organoids. *Cell Rep. Methods* 4:100777. 10.1016/J.CRMETH.2024.100777 38744289 PMC11133841

[B277] Yajuan X. Xin L. Zhiyuan L. (2012). ‘A comparison of the performance and application differences between manual and automated patch-clamp techniques’. *Curr. Chem. Genom.* 6 87–92. 10.2174/1875397301206010087 23346269 PMC3549544

[B278] Yamaura H. Igarashi J. Yamazaki T. (2020). Simulation of a human-scale cerebellar network model on the K computer. *Front. Neuroinform.* 14:16. 10.3389/FNINF.2020.00016 32317955 PMC7146068

[B279] Yamazaki K. Vo-Ho V. K. Bulsara D. Le N. (2022). Spiking neural networks and their applications: a review. *Brain sciences* 12:863. 10.3390/BRAINSCI12070863 35884670 PMC9313413

[B280] Yang N. Chanda S. Marro S. Ng Y. H. Janas J. A. Haag D. (2017). ‘Generation of pure GABAergic neurons by transcription factor programming’. *Nat. Methods* 14 621–628. 10.1038/NMETH.4291 28504679 PMC5567689

[B281] Yang X. Forró C. Li T. L. Miura Y. Zaluska T. J. Tsai C. T. (2024). Kirigami electronics for long-term electrophysiological recording of human neural organoids and assembloids. *Nat. Biotechnol.* 42 1836–1843. 10.1038/S41587-023-02081-3 38253880 PMC11260907

[B282] Yip M. C. Gonzalez M. M. Lewallen C. F. Landry C. R. Kolb I. Yang B. (2024). Patch-walking: coordinated multi-pipette patch clamp for efficiently finding synaptic connections. *bioRxiv [Preprint]* 10.1101/2024.03.30.587445 39556439 PMC11573346

[B283] Yip M. C. Gonzalez M. M. Valenta C. R. Rowan M. J. M. Forest C. R. (2021). Deep learning-based real-time detection of neurons in brain slices for in vitro physiology. *Sci. Rep.* 11:6065. 10.1038/S41598-021-85695-4 33727679 PMC7971045

[B284] Yokoi R. Nagafuku N. Ishibashi Y. Matsuda N. Suzuki I. (2023). ‘Contraindicated drug responses in dravet syndrome brain organoids utilizing micro electrode array assessment methods’. *Organoids* 2 177–191. 10.3390/ORGANOIDS2040014

[B285] Yoon S. J. Elahi L. S. Pas̨ca A. M. Marton R. M. Gordon A. Revah O. (2019). Reliability of human cortical organoid generation. *Nat. Methods* 16 75–78. 10.1038/S41592-018-0255-0 30573846 PMC6677388

[B286] Zdaniuk G. Wierzba-Bobrowicz T. Szpak G. M. Stępień T. (2011). ‘Astroglia disturbances during development of the central nervous system in fetuses with Down’s syndrome’. *Folia Neuropathol.* 49 109–114. 21845539

[B287] Zhang A. Sokolova I. Domissy A. Davis J. Rao L. Hana Utami K. (2022). ‘Maturation delay of human GABAergic neurogenesis in fragile X syndrome pluripotent stem cells’. *Stem Cells Transl. Med.* 11 613–629. 10.1093/STCLTM/SZAC022 35556144 PMC9216490

[B288] Zhang P. W. Haidet-Phillips A. M. Pham J. T. Lee Y. Huo Y. Tienari P. J. (2016). ‘Generation of GFAP::GFP astrocyte reporter lines from human adult fibroblast-derived iPS cells using zinc-finger nuclease technology’. *Glia* 64 63–75. 10.1002/GLIA.22903 26295203 PMC4715664

[B289] Zhang Y. Pak C. H. Han Y. Ahlenius H. Zhang Z. Chanda S. (2013). ‘Rapid single-step induction of functional neurons from human pluripotent stem cells’. *Neuron* 78 785–798. 10.1016/J.NEURON.2013.05.029 23764284 PMC3751803

[B290] Zhang Y. Rózsa M. Liang Y. Bushey D. Wei Z. Zheng J. (2023a). ‘Fast and sensitive GCaMP calcium indicators for imaging neural populations’. *Nature* 615 884–891. 10.1038/S41586-023-05828-9 36922596 PMC10060165

[B291] Zhang Y. Zhang G. Han X. Wu J. Li Z. Li X. (2023b). ‘Rapid detection of neurons in widefield calcium imaging datasets after training with synthetic data’. *Nat. Methods* 20 747–754. 10.1038/S41592-023-01838-7 37002377 PMC10172132

[B292] Zhang Z. Roberson D. P. Kotoda M. Boivin B. Bohnslav J. P. González-Cano R. (2022). ‘Automated preclinical detection of mechanical pain hypersensitivity and analgesia’. *Pain* 163 2326–2336. 10.1097/J.PAIN.0000000000002680 35543646 PMC9649838

[B293] Zhou Z. Yip H. M. Tsimring K. Sur M. Ip J. P. K. Tin C. (2023). Effective and efficient neural networks for spike inference from in vivo calcium imaging. *Cell Rep. Methods* 3:100462. 10.1016/J.CRMETH.2023.100462 37323579 PMC10261900

[B294] Zhu K. Bendl J. Rahman S. Vicari J. M. Coleman C. Clarence T. (2023). Multi-omic profiling of the developing human cerebral cortex at the single-cell level. *Sci. Adv.* 9:eadg3754. 10.1126/SCIADV.ADG3754 37824614 PMC10569714

[B295] Zhu M. H. Jang J. Milosevic M. M. Antic S. D. (2021). Population imaging discrepancies between a genetically-encoded calcium indicator (GECI) versus a genetically-encoded voltage indicator (GEVI). *Sci. Rep.* 11:5295. 10.1038/S41598-021-84651-6 33674659 PMC7935943

[B296] Zlatic M. Robbins M. Christensen C. N. Kaminski C. F. (2021). Calcium imaging analysis - how far have we come? *F1000Research* 10:258. 10.12688/F1000RESEARCH.51755.2 34504683 PMC8406438

